# Sharing DNA-binding information across structurally similar proteins enables accurate specificity determination

**DOI:** 10.1093/nar/gkz1087

**Published:** 2019-11-28

**Authors:** Joshua L Wetzel, Mona Singh

**Affiliations:** 1 The Lewis-Sigler Institute for Integrative Genomics, Princeton, NJ 08544, USA; 2 Department of Computer Science, Princeton University, Princeton, NJ 08544, USA

## Abstract

We are now in an era where protein–DNA interactions have been experimentally assayed for thousands of DNA-binding proteins. In order to infer DNA-binding specificities from these data, numerous sophisticated computational methods have been developed. These approaches typically infer DNA-binding specificities by considering interactions for each protein independently, ignoring related and potentially valuable interaction information across other proteins that bind DNA via the same structural domain. Here we introduce a framework for inferring DNA-binding specificities by considering protein–DNA interactions for entire groups of structurally similar proteins simultaneously. We devise both constrained optimization and label propagation algorithms for this task, each balancing observations at the individual protein level against dataset-wide consistency of interaction preferences. We test our approaches on two large, independent Cys_2_His_2_ zinc finger protein–DNA interaction datasets. We demonstrate that jointly inferring specificities within each dataset individually dramatically improves accuracy, leading to increased agreement both between these two datasets and with a fixed external standard. Overall, our results suggest that sharing protein–DNA interaction information across structurally similar proteins is a powerful means to enable accurate inference of DNA-binding specificities.

## INTRODUCTION

Proteins that bind DNA in a sequence-specific manner are involved in a wide range of functions in the cell, from transcriptional regulation to recombination. Comprehensive knowledge of the DNA-binding preferences of these proteins would thus be a great aid in unraveling the molecular underpinnings of these processes. Fortunately, there has been an explosion in high-throughput experimental techniques for determining DNA-binding preferences for proteins (reviews, (1,2)), and DNA-binding specificities are now known for thousands of naturally occurring proteins spanning a variety of species, including human and most model organisms. Still thousands more specificities have been inferred for synthetic variants from select DNA-binding domain (DBD) families (3,4). Altogether, these specificities cover tens of DBD families, and are easily accessible via expansive databases (5–12).

Accompanying these experimental advances, novel computational approaches have enabled the inference of DNA-binding specificities from raw interaction data (see e.g. (13–18)) and have optimized the inferred models’ abilities to detect *in vivo* binding sites (19–22). Generally, though, current approaches for inferring DNA-binding specificities consider only a single protein at a time, despite the knowledge that proteins within the same DBD family tend to interact with their binding sites in similar ways based upon their common underlying protein–DNA structural interaction scaffold (i.e. they have similar underlying DBD-DNA ‘interfaces’) (23–29). Since high-throughput measurements may be less accurate for some proteins than for others, we reasoned that simultaneously considering *all* observed interactions for large groups of proteins while also considering the similarity of their interfaces would lead to more accurate estimation of DNA-binding specificities. Such an approach is of increasing value as DNA-binding interactions are continuing to be rapidly determined and systematic screens of large numbers of variants for a given DBD family are becoming more common (3,4,30,31).

In this article, we introduce a formal computational framework, along with two specific approaches, for jointly inferring DNA-binding specificities of proteins that share similar underlying structural interfaces. Our framework considers all DNA-binding information across a large collection of proteins within a single DBD family simultaneously in either a constrained optimization or label propagation setting. Our formulation balances inferring specificities that reflect experimental observations for individual proteins with rewarding consistency across inferred specificities when considering the proteins’ similar interfaces. To our knowledge, this is the first approach for inferring DNA-binding specificities that simultaneously considers multiple proteins together in the context of their structural interfaces. In principle, our approaches require only that the large collection of DNA-binding information is for proteins from a DBD family that has a well-characterized DBD-DNA interaction scaffold where it is known which amino acid positions of the DBD are likely to contact and specify bases at particular positions within the specificities.

Here, we demonstrate the power of sharing DNA-binding information across structurally similar proteins via comprehensive testing on two recent independent DNA-binding studies spanning thousands of Cys_2_His_2_ zinc finger (C2H2-ZF) DBDs (4,30); C2H2-ZFs are the most abundant DBD family in higher organisms (32). Applying our framework to each of these datasets individually leads to a ∼15% increase in agreement of DNA-binding specificities for proteins shared across the two datasets; this increase in agreement across repeated independent experiments provides broad evidence that jointly inferred specificities are likely closer to ground truth than their individually inferred counterparts. Moreover, we validate the increased accuracy of jointly inferred specificities by showing increased agreement to a smaller external collection of C2H2-ZF specificities determined from lower throughput experimental data. Finally, as proof of principle, we demonstrate the generality of our framework by applying it to infer specificities for Homeodomain DBDs as well. Overall, we present compelling evidence that joint specificity inference is a powerful, general paradigm to increase the accuracy of specificities derived from high-throughout protein–DNA interaction screens.

## MATERIALS AND METHODS

### Overview of approach

Suppose that we have a group of proteins of the same DBD family, and a measure between pairs of proteins that reflects our expectation as to whether their DNA-binding specificities should be similar. Given a corpus of protein–DNA interaction data across these proteins, our method jointly determines their DNA-binding specificities, as opposed to just determining each protein’s DNA-binding specificity individually, as is typically done.

More formally, suppose we have a set of *n* proteins ${\cal A}$ of the same DBD class, and for each $a \in {\cal A}$, we have an initial estimate of its DNA-binding specificity represented as a position-specific weight matrix (PWM) *S*_*a*_ (or alternatively a count matrix *C*_*a*_). In particular, if *k* is the length of the binding site for the protein, *S*_*a*_ is a 4 × *k* matrix where *S*_*a*_[*b*, *j*] (respectively, *C*_*a*_[*b*, *j*]) is the normalized frequency (respectively, count) with which nucleotide *b* is observed in the *j-*th position of the aligned binding sites for protein *a*; *S*_*a*_ or *C*_*a*_ are usually determined by specialized computational approaches designed to analyze data for *a* arising from specific types of experiments (e.g. protein binding microarrays). We note that binding sites of DBDs typically have a fixed, known length; for example, each C2H2-ZF domain binds a 3 or 4 base pair (bp) site.

For each pair of proteins *a* and *a*′ and for each position 1 ≤ *j* ≤ *k* within the binding site, suppose that we have a weight $w$_*j*_(*a*, *a*′) that represents our *a priori* expectation of how similar the DNA-binding specificities for proteins *a* and *a*′ should be at the *j-*th position in their respective PWMs. If there is no reason to expect that two proteins have similar binding preferences at nucleotide position *j*, then $w$_*j*_(*a*, *a*′) = 0, and otherwise 0 < $w$_*j*_(*a*, *a*′) ≤ 1, with higher values indicating a greater expectation that the DNA-binding specificities of these two proteins are similar. Furthermore, we consider these weights normalized on a per-protein basis (i.e., $\sum _{a^{\prime }}{w_j(a,a^{\prime })} = 1$) so that each protein contributes equally in the optimization formulations below. In a following section, we provide one approach to deriving these weights using structural knowledge about the DBD family.

Our goal is to infer for each $a \in {\cal A}$ a revised DNA-binding specificity $\widehat{S}_a$ such that the DNA-binding specificities of the proteins within ${\cal A}$ are informed both by the initial specificity estimates (i.e. as inferred by analyzing the DNA-binding data for each protein individually) and by the expected similarities between specificities for all the proteins in ${\cal A}$ (as specified by the weights $w$). We give three possible formulations of this problem below, and apply the latter two to infer PWMs.

### Formulations

#### Jointly regularized maximum likelihood

In our first formulation, we consider the case where for each protein *a*, we have count data *C*_*a*_. Our formulation corresponds to a maximum likelihood estimation procedure for inferring PWMs $\widehat{S}_a$ for all $a \in {\cal A}$, where the PWMs are jointly regularized using the weights $w$. Due to computational considerations, we are not able to apply this formulation on protein–DNA binding data in practice, but it provides a framework with which to understand our subsequent approaches.

Here, each column *j* in $\widehat{S}_a$ is modeled as a multinomial distribution, and our goal is to simultaneously estimate the parameters for column *j* for all proteins $a \in {\cal A}$. Let *C*_*a*_[ ·, *j*] denote the the count vector for the *j-*th binding site position for DBD instance *a*, and $\widehat{S}_a[\cdot ,j]$ denote the analogous parameters we wish to estimate for the multinomial distribution. Then ${\cal L}\left(\widehat{S}_a[\cdot ,j]{\big \vert } C_a[\cdot ,j]\right) = Pr\left(C_a[\cdot ,j]{\big \vert }\widehat{S}_a[\cdot ,j]\right)$ is the likelihood function and $-\ell \left(\widehat{S}_a[\cdot ,j]{\big \vert } C_a[\cdot ,j]\right) = -\ln \left({\cal L}\left(\widehat{S}_a[\cdot ,j]{\big \vert } C_a[\cdot ,j]\right)\right)$ is the negative log-likelihood function for the data *C*_*a*_[ ·, *j*] under parameters $\widehat{S}_a[\cdot ,j]$. For each position *j* in the binding site, we determine parameters by solving a constrained optimization problem where we balance minimizing the negative log-likelihoods with the inconsistencies in binding preferences among binding specificity columns that we believe should be similar based on $w$. In particular, for each position *j* we solve:

(1) $$\begin{equation*} \begin{split} \min \limits _{\widehat{S}} \text{ } \sum \limits _a & -\ell \left(\widehat{S}_a[\cdot ,j]{\big \vert }C_a[\cdot ,j]\right)\\ + & \beta \sum \limits _{b} \sum \limits _a\sum \limits _{a^\prime } w_j\left(a,a^\prime \right)\left(\widehat{S}_a[b,j] - \widehat{S}_{a^\prime }[b,j]\right)^2\\ \mbox{subject to:}\\ &\sum _{b} \widehat{S}_a[b,j] = 1 \forall \text{ }a \\ & 0 \le \widehat{S}_a[b,j] \le 1 \forall \text{ }(a,b)\\ \end{split} \end{equation*}$$

where *b* is summed across the possible nucleotide outcomes {A, C, G, T} and *a* and *a*′ are each summed over ${\cal A}$.

The constraints ensure that each PWM column in $\widehat{S}$ forms a distribution, and β is a non-negative constant controlling the level of regularization. In particular, β can be set to be zero if we wish to estimate the PWMs individually; in this case, we will obtain the precise maximum likelihood estimates for each individual PWM in $\widehat{S}$. On the other hand, if we wish to share information across the proteins, we can increase the value of β, and the terms in the second summation will smooth agreement across the *j-*th columns of the PWMs according to our expected similarity measure, $w$ (i.e. jointly regularize the parameter estimates for the multinomials).

Although this formulation has a clean probabilistic interpretation, it poses a few difficulties. First, the multinomial likelihood and negative log-likelihood functions contain exponential and logarithmic terms, respectively; non-linear constrained optimization problems are not practically solvable for a large number of parameters. Second, while most experimental techniques can yield counts, not all do. In contrast, there are technology-specific computational methods for extracting PWMs for all experimental techniques, and PWM models allow similar but more tractable formulations for inferring specificities jointly, as explained below.

#### Convex quadratic programming

Our next formulation modifies the maximum likelihood approach by replacing the negative log likelihood terms with squared error terms relating a set of initial PWM estimates *S* to the output estimates $\widehat{S}$. We use a single fixed parameter 0 < α ≤ 1 in the objective function to balance the original per-protein estimates with the dataset-wide consistency of estimates across all proteins under the measure $w$. For each position *j* in the binding site, our optimization is:

(2) $$\begin{align*} \begin{split} \min \limits _{\widehat{S}} \text{ } \alpha \sum \limits _{b} & \sum \limits _a \left(S_a[b,j] - \widehat{S}_a[b,j]\right)^2\\ + & (1 - \alpha ) \sum \limits _{b} \sum \limits _a\sum \limits _{a^\prime } w_j\left(a,a^\prime \right)\left(\widehat{S}_a[b,j] - \widehat{S}_{a^\prime }[b,j]\right)^2\\ \mbox{subject to:}\\ &\sum _{b} \widehat{S}_a[b,j] = 1 \forall \text{ }a \\ & 0 \le \widehat{S}_a[b,j] \le 1 \forall \text{ }(a,b)\\ \end{split} \end{align*}$$

When α = 1, $S = \widehat{S}$, and as α approaches zero, clusters of proteins for which we expect similar DNA-binding behavior with respect to base position *j* will each have highly similar *j*-th PWM columns. In the case that $S_a[b,j] = C_a[b,j]/\sum _{b^\prime }C_a[b^\prime ,j]$ (i.e. the maximum likelihood estimate for *S*_*a*_[*b*, *j*] based on counts), the primary difference between this formulation and the previous one is that parameter smoothing (‘regularization’) occurs after maximum likelihood estimation rather than simultaneously. Since this objective function is quadratic, the constraints are linear, and the objective function’s Hessian matrix is diagonally dominant with a strictly positive diagonal, the optimization problem is a convex quadratic program and the optimal parameters can be found efficiently. In particular, we use the cvxopt Python package to do so.

#### Label propagation

Our third formulation is based on a general and flexible label propagation algorithm called network ‘adsorption,’ that was initially introduced in the context of improving recommender systems (33). Here, in each iteration, the *j-*th column of the PWM for each protein *a* is updated based on its current value and those of the ‘neighboring’ proteins *a*′ (i.e. those with $w$_*j*_(*a*, *a*′) > 0). That is, column *j* of $\widehat{S}_a$ is initially assigned the value of the column *j* of *S*_*a*_. The algorithm then repeatedly updates $\widehat{S}_a$ as a convex combination of *a* and the neighbors of *a*’s current PWMs according to the following update, where *t* indicates the iteration number, until convergence is reached:

(3) $$\begin{equation*} \begin{split} \widehat{S}_a[\cdot ,j]^{(t)} \text{ } \leftarrow \text{ } & \alpha \widehat{S}_a[\cdot ,j]^{(t-1)} \\ + & (1 - \alpha )\sum _{a^\prime }{w_j{(a, a^\prime )}\widehat{S}_{a^\prime }[\cdot ,j]^{(t-1)}} \\ \end{split} \end{equation*}$$

where $\widehat{S}_a[\cdot ,j]$ is the *j-*th column of $\widehat{S}_a$ and 0 < α ≤ 1 is a fixed parameter balancing the current PWM estimate with the amount of smoothing across related PWMs.

### Similarity measure based on structural knowledge

We now describe how we compute a similarity measure for a DBD family (see Supplemental Methods 1.1 and 1.2 for full details). Briefly, we start by extracting all co-complex protein–DNA structures for the DBD family from BioLiP (34) and performing a multiple structural alignment. Our alignment produces a contact frequency matrix *M*, where *M*[*i*, *j*] is the uniqueness-weighted (35) (to account for redundancy of DBDs across co-complex structures) fraction of DBD-DNA co-complex instances in which an amino acid in position *i* of the DBD contacts a base in aligned binding site position *j* (i.e. within 3.6 Å of a non-hydrogen atom of the base). If an amino acid position *i* contacts a base in at least 10% of DBD–DNA co-complex instances, then we consider it as base contacting. Contact frequency matrices inferred for C2H2-ZFs and Homeodomains highlight known specificity-conferring residues (27,36,37) and are in excellent agreement with previous analyses (Supplementary Figure S1). For two C2H2-ZF DBD sequences *a* and *a*′ that differ in more than one base contacting position, we presume no previous expectation of PWM column similarity (i.e. $w$_*j*_(*a*, *a*′) = 0 for all *j*). Similarly, if *a* and *a*′ vary in the DBD position most frequently contacting position *j*, we set $w$_*j*_(*a*, *a*′) = 0. Otherwise, $w$_*j*_(*a*, *a*′) is set proportionally to 1 – *M*[*i*, *j*], where *i* is the varying key DBD position, and normalized on a per protein basis so that $\sum _{a^\prime }w_j(a, a^\prime ) = 1$. For Homeodomains, we modify this approach to allow non-zero edges for pairs differing in up to four base contacting positions (Supplemental Methods 1.6).

For a DBD family, the similarities between all pairs of DBD sequences can also be represented by a set of graphs *G*_*j*_, one for each base position *j*. In this *similarity graph representation*, there is a node for each DBD sequence *a* and directed edges of weights $w$_*j*_(*a*, *a*′) and $w$_*j*_(*a*′, *a*) connecting nodes for DBD instances *a* and *a*′ if they have non-zero expected similarity in base position *j*.

### PWM datasets

We use two independent datasets of DNA-binding specificities, represented as PWMs, for single C2H2-ZF domains as determined by Persikov, Wetzel *et al.* (4) (the PW-2015 dataset) and Najafabadi, Mnaimneh *et al.* (30) (the NM-2015 dataset). We process these data so that DBDs that are identical in the four base contacting amino acid positions (determined as described above and corresponding to the well-known specificity determining positions for C2H2-ZF domains (4,27,30)) are aggregated; we refer to each set of aggregated sequences by its *core sequence* representation, which is the concatenation of these four amino acids. Our initial set of PWMs consists of 7776 and 2599 distinct core sequences from PW-2015 and NM-2015, respectively. Each PWM is 3 bp long, corresponding to the binding site length of a single domain. Within each dataset, we eliminate core sequences *a* such that there is no *a*′ ≠ *a* in that dataset with $w$_*j*_(*a*, *a*′) > 0 for some *j*, leaving 7760 and 2471 distinct core sequences, respectively, with an overlap of 896 distinct core sequences. Finally, we consider a third set of PWMs corresponding to 150 core sequences from the *D. melanogaster* genome assayed earlier in a lower throughput system (38). Details regarding processing these datasets at the level of core sequences are provided in Supplemental Methods 1.3, and topological properties of the similarity graph representations for the two large datasets are provided in Supplementary Figures S2 and S3. Homeodomain PWM datasets and their processing are described in Supplemental Methods 1.4 and 1.5.

### Evaluating the level of agreement across PWMs

Two PWM columns are considered to be in agreement if their Pearson correlation coefficient (PCC) is ≥0.5. We ensured that our analysis is robust to variations in this threshold, as explained in the Results section. PCC is particularly suitable for our analysis due to its insensitivity to information content (IC), as there are substantial differences in overall IC between the two large PWM datasets and there are changes in IC introduced by our procedure.

## RESULTS

### Rewarding within-dataset consistency increases across-dataset agreement

We begin by considering the performance of our quadratic programming formulation (QP), and then show how the label propagation adsorption formulation (LPA) compares to it in a subsequent section (see Comparison of optimization and adsorption approaches). We test our approaches using the PW-2015 and NM-2015 PWM datasets as initial specificities, determined as described in (4,30) and then processed at the core sequence level (see Materials and Methods). We apply QP to each dataset individually, varying the value of the regularization parameter α that controls the amount of information sharing amongst proteins within a dataset between 1 (no information sharing, the initial PWMs) and 0.05 (heavily rewarding within-dataset consistency). For each α setting, we then measure agreement between corresponding PWM columns for 896 core sequences that are present in both datasets. Since these corresponding PWM columns reflect biologically repeated experiments, we expect high agreement; however, we observe that initial specificities agree for only 60% of columns, with a median per-column PCC of 0.76.

**Figure 1.**
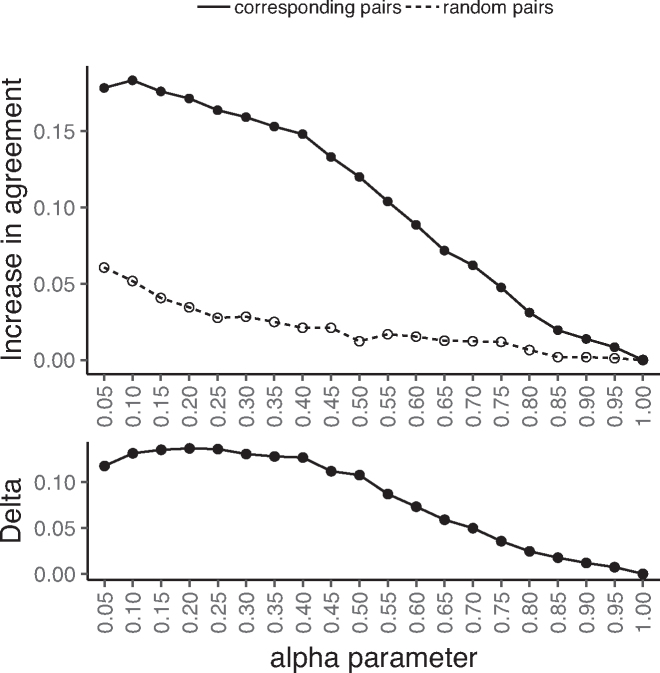
Rewarding within-dataset consistency increases across-dataset agreement. For two independent datasets of single-domain C2H2-ZF specificities, we apply the QP formulation to each dataset separately for different values of α (*x*-axis; lower value implies more information sharing). (Top) For each α, for all proteins shared between the two datasets, we compare their jointly inferred specificities in each of the two datasets and compute the increase in the fraction of corresponding columns in agreement as compared to the agreement between the initial PWMs (solid line; *y*-axis). This increase is substantially larger than when randomly pairing PWMs across the two datasets (dashed line; *y*-axis). (Bottom) As a function of α, we consider the difference in the rate of across-dataset agreement increase for corresponding versus random core sequence pairings (solid line minus dashed line from top panel; *y*-axis), and observe a plateau around α = 0.4 where rates become similar.

Strikingly, as α decreases, the across-dataset agreement increases substantially (Figure 1, top, solid line) as compared to the baseline where there is no joint consideration of proteins (α = 1); this suggests that as information is shared across proteins, each set of inferred PWMs moves independently toward a common ground truth. As a control, we also consider agreement between the PWMs of randomly paired core sequences across the datasets; agreement between random pairs could increase due to protein-independent similarity in background nucleotide distributions across the two datasets. Importantly, for each α < 1 considered, the increase in agreement for the true corresponding PWM columns is far greater than that of the randomly paired columns (Figure 1, top, dashed line), indicating that the increase in agreement for corresponding pairs can not be explained by simple protein-independent similarities in nucleotide backgrounds. Indeed, we find that as α goes from 1 down to 0.4, actual pairings increase in agreement considerably faster than random, after which the difference in rates plateaus (Figure 1, bottom). This suggests that, with smaller α, specificities are rewarded too heavily for consistency with respect to the structural interface. When this plateau at α = 0.4 is reached, the QP approach has led to a 15% increase in agreement for columns of corresponding core sequence pairings across these two datasets, while relatively little increase has occurred for random pairings (2%). These trends are robust to altering the PCC threshold for agreement (Supplementary Figure S4), with median PCCs across paired columns increasing and variances of the PCC distributions decreasing as α decreases (Supplementary Figure S5). Additionally, these same observations hold when considering the individual base positions of the PWMs separately rather than in aggregate (Supplementary Figure S6).

### Initially confident specificities tend not to change

Reasoning that initial specificities reproduced across the two datasets are likely to be correct, we next examine the differential effect of the QP approach on reproduced versus non-reproduced initial specificities. To do so, we partition the corresponding PWM column pairs across the core sequences present in both PW-2015 and NM-2015 into those that initially agree and those that do not (i.e. reproduced and non-reproduced, respectively), and then analyze whether agreement status changes as we reduce α.

**Figure 2.**
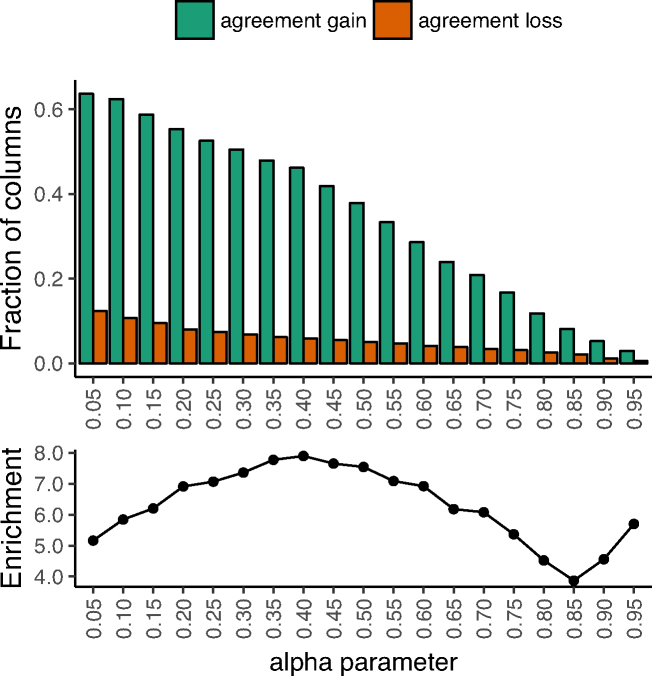
Initially confident specificities tend not to change. For two independent datasets of single-domain C2H2-ZF specificities, we apply the QP formulation to each dataset separately for different values of α (*x*-axis). (Top) For each α, for all proteins shared between the two datasets, we compare their jointly inferred specificities in each of the two datasets and compute the fraction (*y*-axis) of initially disagreeing columns that now agree (green) and the fraction of initially agreeing columns that now disagree (red). For all α, agreement gain is substantially larger than agreement loss. (Bottom) We plot the ratio of these two values (green over red, or the enrichment; *y*-axis), observing 4-to-8-fold enrichment for agreement gain, peaking near α = 0.4. We note that that large enrichments at high α (≥0.90) are an artifact of small sample sizes (i.e. most columns’ agreement statuses have not yet changed from their initial status; see top).

Overall, the fraction of columns in initial disagreement that swap into agreement (‘agreement gain’) vastly exceeds the fraction of columns in initial agreement that swap out of agreement (‘agreement loss’) (Figure 2, top). The ratio of agreement gain to agreement loss is maximized around α = 0.4 (Figure 2, bottom), which coincides with the performance plateau observed in the previous section (Figure 1, bottom). At this ‘optimal’ regularization level, there is ∼8-fold enrichment for agreement gain over loss (46% and 6%, respectively). Thus our approach does not tend to change agreement for specificities reproduced across the two datasets.

For a wide range of α settings, the vast majority of jointly inferred specificities are in good agreement with corresponding initial specificities. For example, at α = 0.4, 92% of columns agree with their corresponding initial counterparts (Supplementary Figure S7). Strikingly, when considering the subset of paired columns (i.e., one from each dataset, corresponding to the same core sequence) from the across-dataset ‘agreement gain’ group, at least one column from each pair remains in agreement with its initial counterpart 99% of the time (Supplementary Figure S8). This suggests that one of the two initial specificities is typically already accurate, and our procedure is highly unlikely to alter that particular one.

As a control, when repeating our QP procedure after randomizing core sequence relationships within each dataset by permuting nodes within each similarity graph, we find that the agreement gain vs. loss ratio remains close to 1 for nearly all α settings tested (Supplementary Figure S9) and corresponding columns across the two datasets decrease in agreement (Supplementary Figure S10). Furthermore, PWM columns inferred under such random core sequence associations lack information content and diversity (Supplementary Figures S11 and S12).

### Comparison with an external dataset validates improvements

**Figure 3.**
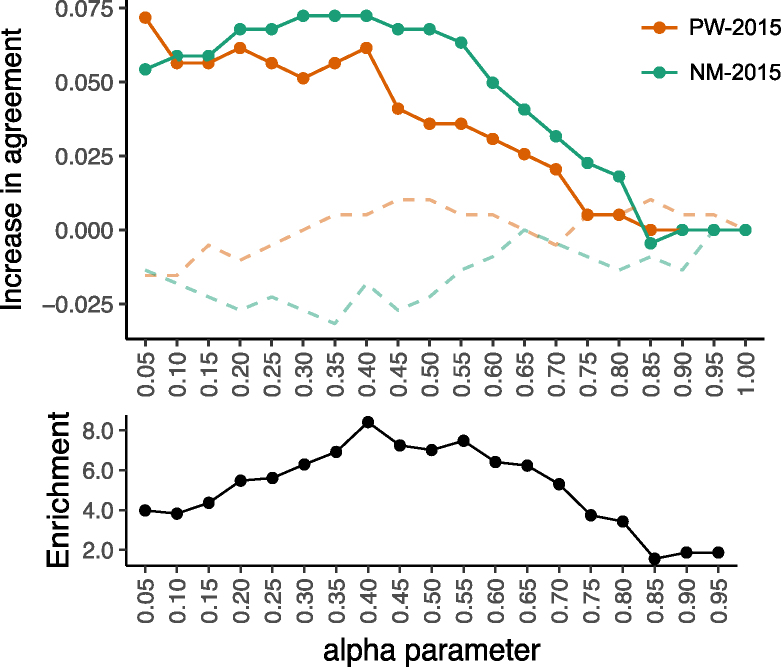
Comparison with an external dataset validates improvements. For the PW-2015 and NM-2015 datasets of single-domain C2H2-ZF specificities, we apply the QP formulation to each dataset separately for different values of α (*x*-axis). (Top) For each α, for each protein shared between PW-2015 and an external dataset (38), we compare the jointly inferred specificity in the PW-2015 dataset with the corresponding specificity in (38) and compute the increase in the fraction of columns in agreement (top, *y*-axis) as compared to the agreement between the initial PWMs (solid red line). Similarly, we compute this same increase in agreement between the jointly inferred specificities for NM-2015 and their corresponding specificities in (38) (solid green line). Agreement with the external dataset increases in both cases as more information is shared among proteins in the same dataset (i.e., by decreasing α), until either a plateau or a peak is reached around α = 0.4. When comparing jointly inferred PWM columns to randomly chosen PWM columns from the external set (dashed lines), little to no agreement increase is observed. (Bottom) We consider the ratio for agreement gain to agreement loss (*y*-axis) at each α setting (analogous to Figure 2, bottom), aggregating columns across both datasets simultaneously, and observe a peak enrichment of ∼8-fold at α = 0.4.

We further evaluate the accuracy of the jointly inferred specificities we derived from PW-2015 and NM-2015 by considering agreement of each with a more reliable dataset of 150 specificities for C2H2-ZF core sequences determined independently from lower throughput data (38). Of these core sequences, 67 and 80 overlap with PW-2015 and NM-2015, respectively. Considering these overlapping core sequences, at α = 0.4 we find that specificities have 6–7% more columns in agreement with the external dataset than do the corresponding initial specificities (Figure 3, top). Furthermore, the curve for ratio of agreement gain to agreement loss is qualitatively similar to that observed in our analysis above, again with peak enrichment for agreement gain of roughly 8-fold at α = 0.4 (Figure 3, bottom). Thus, the increased accuracy suggested by our large-scale analyses is recapitulated when considering this smaller but more reliable dataset. Several examples of jointly inferred specificities as compared to their initial individually inferred counterparts are shown in Supplementary Figure S13.

### Comparison of optimization and adsorption approaches

We repeat the analyses described above using the LPA algorithm (33). In LPA, α corresponds to the probability of entering an absorbing state during a random walk through the transpose of the similarity graph, which differs from its interpretation in the QP approach. Thus we do not necessarily expect similar performance at the same α across the two approaches. Instead we ask whether for each α setting for QP, there exists some α′ for LPA at which the approaches perform similarly.

**Figure 4.**
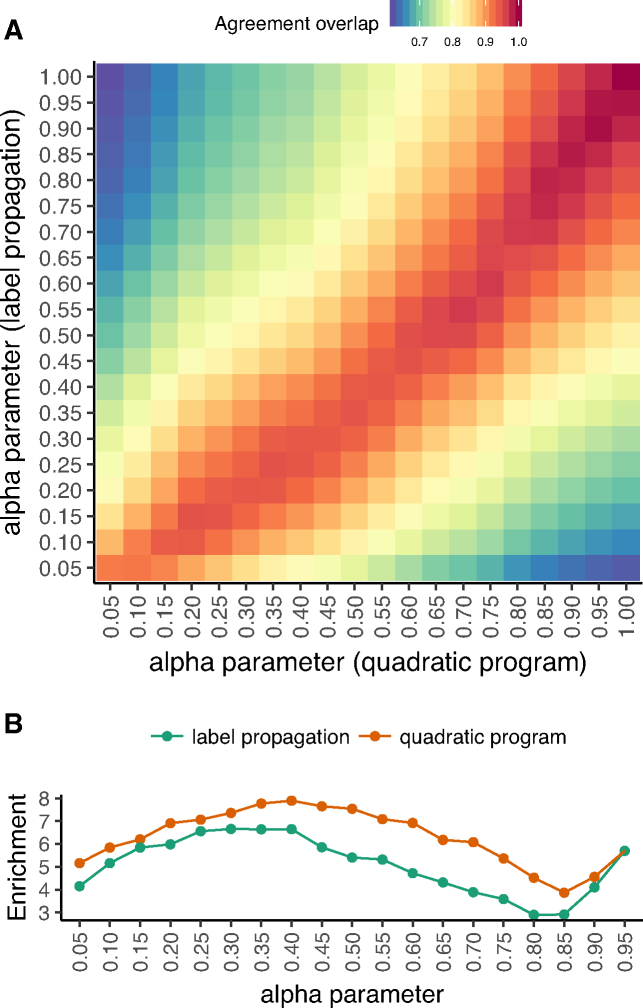
Quadratic programming and label propagation adsorption approaches yield similar jointly determined specificities. We consider the similarity of results between our two distinct approaches to sharing DNA binding information across structurally similar proteins. (**A**) For different values of α for the QP approach (*x*-axis) and for the LPA approach (*y*-axis), we plot the Jaccard coefficient of the sets of corresponding columns in agreement across PW-2015 and NM-2015 (i.e. the agreement overlap). Lower overlap is indicated by blue and higher overlap is indicated by red. (**B**) As a function of α (*x*-axis), we compare the agreement gain versus agreement loss ratio (i.e., as described in Figure 2; *y*-axis) when using either the LPA approach (green) or the QP approach (red, also shown in Figure 2, bottom). The QP approach obtains higher ratios for all α and the ratio peaks at 0.3 $\leq$ α $\leq$ 0.4 for both approaches.

We compare results of the QP and LPA approaches across a grid of (α, α′) settings, computing the Jaccard coefficient overlap of the PWM columns that are in across-dataset agreement for NM-2015 and PW-2015. The two approaches produce highly similar, though non-identical, across-dataset agreement profiles, as indicated by the slightly asymmetric red diagonal in Figure 4 A (corresponding to Jaccard coefficient > 0.9). When considering each approach at its best α setting according to the agreement gain to agreement loss ratio, QP performs slightly better than LPA (7.9-fold at α = 0.4 versus 6.7-fold at α = 0.3, respectively; see Figure 4 B). At these α settings, the overlap in sets of columns in across-dataset agreement is 0.94, and the increase in fraction of PWM columns in across-dataset agreement is similar (15% for QP and 13% for LPA). Overall, the same general trends observed when using QP are preserved when switching to LPA (Supplementary Figures S14 and S15), with LPA performing slightly worse but at lower computational cost.

Since label propagation is a natural extension of a nearest neighbor (NN) approach, we perform additional testing to ensure that LPA outperforms an analogous NN approach. Specifically, we repeat our analysis, but run LPA for only a single iteration, which is identical to weighted NN using the same structural similarity measure. LPA to convergence substantially outperforms NN, both in terms of increase in across-dataset agreement between NM-2015 and PW-2015 and each datasets’ agreement with the smaller external dataset (38) (Supplementary Figure S14).

### Application to Homeodomains

As a proof of principle demonstration of the generality of our approach, we next apply it to infer the DNA-binding specificities of Homeodomain proteins. Homeodomains comprise the second most abundant class of transcription factors in humans (32), and more generally account for an estimated 15-30% of transcription factors across plants and animals (39). While Homeodomains generally bind DNA via a 6–8 bp long region, PWMs extracted from the *cis*-BP (8) database for Homeodomains vary in length. We thus aligned each of 429 Homeodomain PWMs extracted from *cis*-BP to their appropriate positions within our structural contact model for Homeodomains (Supplemental Methods 1.4 and 1.5; Supplementary Figure S1, bottom). These PWMs span 314 distinct proteins, of which 231 have a single PWM, while the remaining each have up to four ‘replicate’ PWMs from separate publications. In general, specificities for distinct Homeodomain proteins are less diverse than those for ZFs, with the majority of Homeodomains’ PWMs containing a TAAT motif in the first four of six ‘core’ binding site positions. We find that the replicate PWMs have excellent agreement in these four positions (labeled 1 through 4 in Supplementary Figure S16). However, there are some disagreements at positions 5 and 6, and thus we apply our procedure for jointly inferring DNA-binding specificities in order to determine whether we can obtain higher agreement for these positions.

After randomly partitioning the PWMs into two sets of roughly equal size, with 83 proteins represented by replicate PWMs in opposite sets, we apply our QP approach to positions 5 and 6 of each set independently at various α settings (Supplementary Results 2.1 and Supplementary Methods 1.6). Of the corresponding column pairs across the two sets that disagree initially (i.e. at α = 1), 61% gain agreement at α ≤ 0.7, while none of the initially agreeing columns lose agreement (Supplementary Figure S17, top left). This agreement gain far exceeds that observed for columns randomly paired across the two sets (18% at α = 0.7; Supplementary Figure S17, bottom left). Overall, sharing knowledge across proteins tends to result in higher PCCs between corresponding columns; for example, at α = 0.7, 66.4% of paired columns have increased PCCs, 30.3% have PCCs that are the same, and only 3.3% have decreased PCCs (Supplementary Figure S17, right). Thus, even in a challenging testing scenario where specificities are highly accurate to start, our framework increases reproducibility of PWM estimates across independent experiments. Visual examples of improved agreement for Homeodomain specificities are provided in Supplementary Figure S18.

## DISCUSSION

Here, we have introduced a general framework for DNA-binding specificity estimation that simultaneously considers interaction preferences for an entire group of proteins from the same DBD family. At the heart of our framework is the notion of rewarding global consistency of specificities according to an expected similarity measure that reflects DBD family-level structural considerations. We have shown several lines of evidence supporting the advantages of our framework over simply estimating each specificity individually. First, determining specificities jointly substantially improves across-dataset agreement for two large-scale, independent studies. Second, this approach rarely perturbs reliable and reproducible initial specificities, instead selectively correcting less confident ones. Third, we verified that the specificities jointly determined based on either of the two large-scale datasets are in better agreement with a reliable external set than the corresponding initial specificities are.

The framework we have described here is technology-independent and designed to be applied as a complementary post-processing step to any sufficiently large set of PWMs for proteins that share similar underlying structural DBD-DNA interfaces. Indeed, we have used our framework to infer Homeodomain DNA-binding specificities that consider measurements from across multiple experimental platforms simultaneously. Much previous work improving specificities derived from high-throughput protein–DNA interactions has been technology-specific, as the relationship between actual binding events and measured signals of binding is itself technology-specific. For example, a competition comparing over twenty algorithms highlighted this inherent challenge in the context of predicting probe intensities for protein binding microarrays (PBMs) (16). As technology-specific approaches continue to advance models relating raw signal to specificities of individual proteins, our joint framework can leverage these improved models.

One potential limitation of our approach is that it requires knowledge of the structural interface between an instance of a DBD and its binding sites, as represented by a PWM. This interface can be inferred, even from limited structural examples, either by hierarchically aligning sufficiently similar PWMs across distinct DBD instances (40), or via specialized experimental setups that directly provide position and orientation information across all the detected DBD-DNA interactions (4,30,36). For example, here we use a heuristic approach based upon a limited set of known interfaces (36) as well as the similarity of key base-contacting DBD residues to align Homeodomain DBD–PWM pairs to underlying structural interfaces (Supplemental Methods 1.5). Given the breadth of co-complex structures available in the PDB (41,42), we expect that further development of algorithms for jointly determining DNA-binding specificities may be able to automatically infer the necessary structural interfaces from more general experimental data (i.e. with unknown registration of PWM positions across proteins), even for DBD families with complex and diverse binding preferences. Alternatively, previously correlations between DBD residues and bound DNA sequences for a particular DBD family have been leveraged to infer models predictive of changes in DNA-binding specificity (43) or even family-wide recognition codes (25,30,44–47); it may be that such correlations can also be harnessed to guide sharing of information across structural interfaces of DBD–PWM pairs.

Our approaches allow flexibility in the amount of DNA-binding information that is shared across proteins within a dataset via a single tunable parameter, α. In many settings, independent datasets include specificities for overlapping sets of proteins. In this case, we have shown already that several measures of improvement—including overall increase in across-dataset agreement relative to a null model (Figure 1, bottom) and enrichment for agreement gain over agreement loss (Figures 2 and 3, bottom)—are very helpful for choosing the α parameter. If substantially overlapping datasets are not available, we recommend considering the fit of the initial PWMs to the underlying data that is being modeled. For example, α can be tuned to allow some information sharing (e.g. 0.5 ≤ α ≤ 0.9) while also requiring that well-fitting initial PWMs from sufficient data should be minimally perturbed. Importantly, we note that precise tuning of the parameter is not strictly required; indeed we have shown that even small amounts of information sharing across proteins (i.e. large α) can substantially improve PWM estimates.

While our framework is developed in the regime of classical PWMs, more complex models of specificity relax probabilistic base position independence assumptions inherent to classical PWMs. This is done, for example, via regression on DNA *k*-mer features (14,16,17,48), direct inclusion of DNA-shape information (18,20–22), and/or direct formulation of specificity models in terms of binding energy estimates (13,49–51)). One advantage of formulating in terms of classical PWMs is the ability of our framework to accommodate DNA-binding specificities derived from various sources; complex models can be converted to simpler ones under basic independence and/or scaling assumptions. While our approach already improves specificities substantially, we anticipate that extending it to allow inter-base dependencies may lead to even higher improvements for select proteins, albeit at the expense of additional parameters.

In sum, protein–DNA interactions continue to be rapidly determined in the laboratory, and assays considering large numbers of variants of DNA-binding proteins of the same DBD family are becoming commonplace. Here we have presented a general framework for joint PWM inference that allows simultaneous consideration of entire groups of structurally similar DNA-binding proteins during specificity determination. We have demonstrated that an existing label propagation algorithm can provide comparable results to directly optimizing an objective, as the fundamental concept of encouraging consistency across specificity estimates for similar DBD instances is reflected in either formulation. In the future, alternate optimizations of the joint PWM inference problem under various algorithmic or statistical objectives is likely to extend the capabilities of the framework and to lead to even more accurate estimates of DNA-binding specificity.

## Supplementary Material

gkz1087_Supplemental_File
