# Supplementary material for: Sharing DNA-binding information across structurally similar proteins enables accurate specificity determination
Source: Nucleic Acids Res. 2019 Nov 28;48(2):e9. doi: 10.1093/nar/gkz1087 (PMC7028011; doi:10.1093/nar/gkz1087)
Supplement: gkz1087_Supplemental_File [file gkz1087_supplemental_file.pdf]

Sharing DNA-binding information across structurally similar proteins enables accurate specificity determination (Supplemental Materials)

Joshua Wetzel and Mona Singh

1 Supplemental Methods

1.1 Alignment of co-complex structural data

In order to allow computation of pairwise expected similarity between proteins' DNA-binding specificities, we inferred position-specific contact frequency models from protein-DNA co-complex structures for C2H2-ZFs and Homeodomains. Briefly, our algorithm performs multiple co-complex structural alignment across co-complex structures from the same DBD family and aggregates contact information between DBD positions and binding site positions based on that alignment; finally the algorithm outputs a model, D, where $D[i, j]$ corresponds to the fraction of times that base-contacting DBD position i physically contacts "reference" binding site position j across a collection of DBD-DNA co-complex structures.

In particular, for each DBD–DNA co-complex k that has l_k DNA base pairs, we create an $h \times l_k$ dimensional matrix C_k, where h is the number of match states in an HMM (e.g., from PFAM [1]) for the corresponding DBD family. We set $C_k[i, j] = 1$ if the j-th DNA base is the closest base to the amino acid in the i-th match state and is in contact with it (as defined in the following section), and 0 otherwise. Each matrix C_k may have a different number of columns (DNA positions), and we wish to find a common offset and orientation (i.e., registration) across all the matrices. Intuitively, our algorithm finds this registration by horizontally translating and/or flipping each C_k such that the total number of non-zero cells when summing the matrices (under the registration) is as small as possible. We note that searching the space of all combinations of orientations and offsets is computationally infeasible; thus we employ a greedy heuristic strategy (as is frequently done in multiple sequence alignment). In practice, for both our alignment procedure and calculation of the fraction of times each DBD position contacts a base in a particular position of the alignment, we down-weight contributions from subsets of DBD instances that are similar to each other [2, 3].

Concretely, for each DBD–DNA co-complex k, denote the horizontal offset by $a_k > 0$ and the flip direction by $b_k \in \{1, -1\}$. We begin by trimming off columns from either end of each matrix, C_k, if no amino acids contact the corresponding base positions. We then initialize an *aggregate* contact matrix D to be the contact matrix with the widest dimension after trimming. All other matrices will be aligned relative to this one, so that j-th position in this matrix gets mapped to $j + a_k * b_k$ in matrix C_k. For each remaining contact matrix, C_k, iteratively, and in arbitrary order, we perform the following two steps: 1. Find the shift a_k and flip direction b_k that maximizes the Frobenius inner product of D and C_k after padding C_k with zero columns to be the same width as D. 2. Update the value of D by adding its current value to the padded version of C_k corresponding to the maximizing shift/orientation. Finally, positions in D are manually oriented relative to a well understood structure for the DBD family to obtain the "reference" base numbering scheme.

1.2 Preprocessing protein-DNA co-complex structures

All co-complex structures from the BioLIP database [4] for biologically relevant protein-ligand interactions from the Protein Data Bank [5] were downloaded and subsequently peptide chains were searched for DBD instances using probabilistic HMM matching [1], as described in previous work [3]. Subsequently, each DBD instance in any peptide chain was assigned to its closest paired homologous DNA ligand chains, thus creating a set of DBD-DNA pairs. We excluded DBD instances prior to our co-complex alignment procedure (i.e., for Section 1.1) based on two possible criteria: First, since our alignment algorithm assumes each DBD instance to have a fixed number of match states, DBD instances with missing match states were removed. Second since we are interested in interactions with double-stranded DNA, we removed DBD instances whose closest matched DNA ligand did not have a corresponding homologous strand over the region where it interacts with the DBD instance. Overall, a total of 287 C2H2-ZF and 73 Homeodomain instances remained after removing instances due to either of these criteria. For each DBD-DNA pair in the remaining set, the bases in the DNA chain were numbered in order choosing one of the two strands to be the forward strand arbitrarily, then contacts between side-chains marked as corresponding to an HMM match state and DNA-base positions were recorded, separating into base-contacts and backbone-contacts. For this work, we defined a 'contact' as a heavy atom (i.e., non-hydrogen) of an amino acid side chain within a distance of 3.6Å of a heavy base or backbone atom; this cutoff captures hydrogen bonds and van der Waals interactions, but not water-mediated interactions [6].

1.3 Processing C2H2-ZF PWM datasets at the core sequence level

Initial PWMs for the `PW-2015` dataset were inferred from bacterial-one-hybrid (B1H) selections described in Persikov, Wetzel, *et al.* [7]. For the analyses described here, we considered only the subset of B1H selections performed at low stringency (2mM) of the interaction inhibiter 3-aminotriazole. These selection data were processed essentially as described in our previous work [7], with a few minor exceptions. First, C2H2-ZFs with only one possible encoding at the nucleotide level were removed from all selections. Second, constructs encoding identical C2H2-ZFs at the amino acid level were aggregated based on the mean observed frequency across nucleotide variants within a selection rather than by the sum across variants. Third, when averaging frequencies of identical C2H2-ZFs across the F2 and F3 selections (i.e., positions of the randomized C2H2-ZF within construct, see [7]), frequencies of C2H2-ZFs that did not pass our entropy threshold (see [7]) in one selection but did pass in the other were 'rescued' for the selection where they did not pass the threshold, prior to averaging. As in our previous work, these average frequencies (i.e., across F2 and F3) were then aggregated at the 'core sequence' level (i.e., positions -1, 2, 3, and 6 relative to the start of the DNA-contacting α-helix according to the structural interface) and used as input to a 'lookup' procedure [7], which produced 3 bp PWMs corresponding to 7,776 distinct C2H2-ZF core sequences.

Initial PWMs for the `NM-2015` dataset were derived from matrices corresponding to relative free energies of binding to each nucleotide as reported by Najafabadi, Mnaimneh *et al.* [8]. For each of the 8,138 C2H2-ZFs with reported energy matrices, we scanned the reported protein sequence constructs to determine unambiguous positions -1 through 6 of the α-helix via regular expression matching, requiring no insertions or gaps within the α-helix. For the 8,129 C2H2-ZFs that matched the regular expression, per-base-position energies were converted to per-base-position probabilities (i.e., PWM columns) by taking the exponential of energies for each base in a given position then normalizing to distribution. This corresponds directly to per-base position probabilities arising from a Boltzmann distribution with the corresponding free energies and an assumed exponential scaling factor of 1. Since the energies are unscaled (personal communication with the first author of [8]) any chosen scaling factor will be somewhat arbitrary. When visualizing as logos, PWMs were rescaled to match the average per-column information

content of an external reference [9], using a procedure previously described by Christensen et al. [10]. PWMs of C2H2-ZFs from this dataset identical in their core sequence positions were averaged per base position, resulting in a single PWM for each of 2,599 distinct core sequences.

Finally, as an external standard, we considered the 238 C2H2-ZF PWMs provided as Supplemental Dataset 1 from the work of Enuameh, Asriyan, *et al.* [9], where specificities were determined in a lower throughput system, and individual C2H2-ZF domains from full-length fly proteins were assigned manually by the authors to their corresponding binding subsites. Again, PWMs of C2H2-ZFs identical in their core sequence positions were averaged per base position, resulting in a single PWM for each of 150 distinct core sequences.

1.4 Gathering Homeodomain DNA-binding specificities

We extracted 612 DNA-binding specificities (in the form of PWMs) spanning 395 distinct Homeodomain (HD) TFs from the Cis-BP database [11]. In particular, we considered PWMs of proteins naturally occurring in human, mouse, or fly that were annotated as containing a HD DBD and no other DBD type, and that had been assayed *in vitro* via protein-binding microarray, SELEX, or bacterial one-hybrid assays spanning 8 distinct publications [12, 13, 14, 15, 16, 17, 18, 11]. After excluding PWMs that corresponded to binding in the presence of methylcytosine or mutational analyses we arbitrarily chose one motif for each TF assayed in each study to consider for further analysis. In the case of one study by Yin et al. [16], binding was assayed for some TFs expressed either as full-length proteins or as "extended DBDs"; here we gave preference to the extended DBD versions as they covered a larger number of total distinct TFs and were previously demonstrated to be highly similar to the full-length versions in most cases. In the case of motifs from FlyFactorSurvey [17], we preferentially used motifs derived from Solexa sequencing of binding sites, if available.

1.5 Alignment of Homeodomain PWMS to reference base positions

We aligned the HD PWMs extracted from the Cis-BP database [11] to "reference" base positions within our structural contact model for HDs (see Supplemental Methods 1.1 and 1.2; Supplemental Figure S1). While mappings between PWM positions and bases within a contact model are not known *a priori*, here we inferred mappings using similarity of key base-contacting residues to 84 distinct HD proteins in fly for which such mappings were determined experimentally [19]. PWMs from these proteins clustered into 11 distinct "specificity groups", and group membership was well correlated with the identities of amino acids occupying DNA-contacting DBD positions [19].

Specifically, for each of the HD PWMs in our collection (Supplemental Methods 1.4), we first searched its corresponding protein sequence (extracted from Uniprot [20]) for an HD instance using HMMer v.3.1.b2 [21] with the default HD gathering thresholds. We assigned each protein a "fingerprint" by concatenating amino acids occupying base-contacting DBD positions (as defined in the main manuscript). Each PWM was assigned to one of the 11 fly specificity groups based on highest percent amino acid identity of its fingerprint to any of the 84 fly HD proteins with known mappings, then aligned to an "exemplar" PWM from its assigned specificity group by minimizing mean squared error across aligned column pairs. In order to ensure confident mappings, we only considered alignments where the consensus sequence of the newly aligned PWM matched a regular expression corresponding to a key pattern within the "core" HD binding site (e.g., 'TAAT' in positions 1 through 4 for proteins assigned to the Engrailed specificity group, 'T/CAAG' in positions 1 through 4 for the NK group, etc.). If no such alignment could be found, the PWM was excluded from further analysis. Furthermore, PWMs were excluded if the corresponding DBD was missing fingerprint match states from the HD HMM or if we failed to find an unambiguous mapping from the gene name to a valid Uniprot ID.

1.6 Structure-based similarity scores for Homeodomains

We extend the structure-aware similarity measure described in the main manuscript to compute expected pairwise similarity scores for reference base positions 5 and 6 of the HD DBD instances, allowing up to 4 varying base-contacting DBD positions between HD protein pairs. In particular, for any pair of HD proteins, a and a', we defined $w(a, a') = 0$ if a and a' were non-identical in any of the DBD positions 47, 50, and 54, which are the most likely DBD positions to specify a base in position 5 or 6 according to previous literature [19] and according to our deduced contact model (see Supplemental Figure S1). Additionally, neighboring proteins were required to be at least 85% similar in their remaining fingerprint positions according to a scaled BLOSUM 62 score, with $w(a, a')$ set proportionally to this score. More precisely, we set $w(a, a') = \max\{0, f(a, a')/\max\{f(a, a), f(a', a')\} - 0.85\}$, where $f(x, y) = \sum_i BL62(x_i, y_i)$ across all remaining DBD fingerprint positions i, and $BL62$ is the BLOSUM 62 amino acid substitution scoring matrix. These weights are normalized on a per protein basis so that $\sum_{a'} w_j(a, a') = 1$.

2 Supplemental Results

2.1 Jointly inferring HD PWMs improves across-dataset agreement

Using the alignment procedure outlined above (Supplemental Methods 1.4 and 1.5), confident base position mappings to a structural contact matrix for the HD DBD family (see Supplemental Figure S1) were inferred for 429 HD PWMs extracted from the Cis-BP database. These PWMs spanned 314 distinct HD proteins, of which 231 had a single mapped PWM. Of the remaining proteins, 54, 26, and three had two, three, and four mapped PWMs, respectively, from independent publications (herein referred to as the "replicate" set). We performed pairwise comparisons of PWM columns for corresponding proteins within this replicate set, including one base position upstream and one downstream of the six bp "core" HD binding site as defined previously in Noyes et al. [19]. Aggregate analysis and visual inspection of the PWMs indicated excellent agreement for the first four core motif positions (i.e., "TAAT" consensus positions for the Engrailed and Antp subfamilies; labeled 1 through 4 in Supplemental Figure S16), indicating overall accuracy of the inferred base position mappings as well as a high degree of reproducibility across publications. However, we observed some disagreement for corresponding columns in the last two core motif positions (5 and 6), where base preferences have been previously correlated with specific DBD residue combinations in positions 47, 50, and 54 of the HD recognition helix [19, 12]. While disagreement was also observed for flanking positions 0 and 7, these positions contribute only weakly to specificity and their specificity determinants are poorly understood.

Based on these findings, we focused a proof-of-principle experiment to determine whether our joint PWM estimation approach could improve concordance of positions 5 and 6 across replicates. To do so, we partitioned the set of HD PWMs into two hypothetical independent datasets, A and B: Each HD protein that had only a single PWM in our set was first placed uniformly at random into either A or B, and then each HD in the replicate set had its independently derived PWM instances stratified uniformly at random across A and B. This resulted in A and B containing 214 and 215 motifs, respectively, allowing a total of 118 pairwise comparisons, per base position, for PWMs corresponding to the same protein that lie on opposite sides of the partition. Overall, initial agreement of corresponding columns pairs for positions 5 and 6 across sets A and B is quite high, with 218 out of 236 (92%) in good agreement (PCC ≥ 0.50). We applied our QP approach to PWM columns from A and B independently using a simple structure-based *a priori* expected similarity measure (considering base positions 5 and 6 only, Supplemental Methods 1.6) and at various α settings. Remarkably, using $\alpha < 1$ in our QP formulation (i.e., introducing prior information regarding expected relationships between

proteins' specificities based on structural knowledge) improves agreement across corresponding pairs even further, with 11 out of 18 initially disagreeing column pairs (61%) moving into agreement for $\alpha \leq 0.7$ (Supplemental Figure S17, top left). Moreover, none of the 218 columns that initially agreed moved into disagreement for $\alpha < 1$. Overall, sharing knowledge across proteins resulted in higher PCCs between corresponding columns; for example, at $\alpha = 0.7$, 66.4% of paired columns increase in raw PCC agreement, 30.3% do not change in PCC, and only 3.3% decrease in PCC (Supplemental Figure S17, top right). Columns randomly paired across A and B display substantially less agreement gain than corresponding pairs at any α setting (18% at $\alpha = 0.7$; Supplemental Figure S17, bottom left) and are substantially more likely to decrease in raw PCC score as α is lowered (e.g., 28% vs. 3% for random vs. corresponding pairs at $\alpha = 0.7$, respectively; Supplemental Figure S17, right). We provide visual examples for increased agreement of PWMs in Supplemental Figure S18.

References

[1] Finn, R. D., Bateman, A., Clements, J., Coggill, P., Eberhardt, R. Y., Eddy, S. R., Heger, A., Hetherington, K., Holm, L., Mistry, J., Sonnhammer, E. L., Tate, J., and Punta, M. (jan, 2014) Pfam: The protein families database. *Nucleic Acids Research,* **42**(D1), D290–301.

[2] Henikoff, S. and Henikoff, J. G. (1994) Position-based sequence weights. *Journal of Molecular Biology,* **243**(4), 574–578.

[3] Kobren, S. N. and Singh, M. (2019) Systematic domain-based aggregation of protein structures highlights DNA-, RNA-, and other ligand-binding positions. *Nucleic Acids Research,* **47**(2), 582–593.

[4] Yang, J., Roy, A., and Zhang, Y. (2013) BioLiP: A semi-manually curated database for biologically relevant ligand-protein interactions. *Nucleic Acids Research,* **41**(D1), D1096–103.

[5] Berman, H., Henrick, K., and Nakamura, H. (2003) Announcing the worldwide Protein Data Bank. *Nature Structural Biology,* **10**(12), 980–980.

[6] Persikov, A. V. and Singh, M. (jun, 2011) An expanded binding model for Cys2His2 zinc finger protein-DNA interfaces. *Physical Biology,* **8**(3), 35010.

[7] Persikov, A. V., Wetzel, J. L., Rowland, E. F., Oakes, B. L., Xu, D. J., Singh, M., and Noyes, M. B. (2015) A systematic survey of the Cys2His2 zinc finger DNA-binding landscape. *Nucleic Acids Research,* **43**(3), 1965–1984.

[8] Najafabadi, H. S., Mnaimneh, S., Schmitges, F. W., Garton, M., Lam, K. N., Yang, A., Albu, M., Weirauch, M. T., Radovani, E., Kim, P. M., Greenblatt, J., Frey, B. J., and Hughes, T. R. (2015) C2H2 zinc finger proteins greatly expand the human regulatory lexicon. *Nature Biotechnology,* **33**(5), 555–562.

[9] Enuameh, M. S., Asriyan, Y., Richards, A., Christensen, R. G., Hall, V. L., Kazemian, M., Zhu, C., Pham, H., Cheng, Q., Blatti, C., Brasefield, J. A., Basciotta, M. D., Ou, J., McNulty, J. C., Zhu, L. J., Celniker, S. E., Sinha, S., Stormo, G. D., Brodsky, M. H., and Wolfe, S. A. (jun, 2013) Global analysis of Drosophila Cys2-His2 zinc finger proteins reveals a multitude of novel recognition motifs and binding determinants. *Genome Research,* **23**(6), 928–940.

[10] Christensen, R. G., Enuameh, M. S., Noyes, M. B., Brodsky, M. H., Wolfe, S. A., and Stormo, G. D. (2012) Recognition models to predict DNA-binding specificities of homeodomain proteins. *Bioinformatics,* **28**(12), i84–9.

[11] Weirauch, M. T., Yang, A., Albu, M., Cote, A. G., Montenegro-Montero, A., Drewe, P., Najafabadi, H. S., Lambert, S. A., Mann, I., Cook, K., Zheng, H., Goity, A., van Bakel, H., Lozano, J.-C., Galli, M., Lewsey, M. G., Huang, E., Mukherjee, T., Chen, X., Reece-Hoyes, J. S., Govindarajan, S., Shaulsky, G., Walhout, A. J., Bouget, F.-Y., Ratsch, G., Larrondo, L. F., Ecker, J. R., and Hughes, T. R. (2014) Determination and Inference of Eukaryotic Transcription Factor Sequence Specificity. *Cell,* **158**(6), 1431–1443.

[12] Berger, M. F., Badis, G., Gehrke, A. R., Talukder, S., Philippakis, A. A., Peña-Castillo, L., Alleyne, T. M., Mnaimneh, S., Botvinnik, O. B., Chan, E. T., Khalid, F., Zhang, W., Newburger, D., Jaeger, S. A., Morris, Q. D., Bulyk, M. L., and Hughes, T. R. (2008) Variation in Homeodomain DNA Binding Revealed by High-Resolution Analysis of Sequence Preferences. *Cell,* **133**(7), 1266–1276.

[13] Barrera, L. A., Vedenko, A., Kurland, J. V., Rogers, J. M., Gisselbrecht, S. S., Rossin, E. J., Woodard, J., Mariani, L., Kock, K. H., Inukai, S., Siggers, T., Shokri, L., Gordân, R., Sahni, N., Cotsapas, C., Hao, T., Yi, S., Kellis, M., Daly, M. J., Vidal, M., Hill, D. E., and Bulyk, M. L. (2016) Survey of variation in human transcription factors reveals prevalent DNA binding changes. *Science,* **351**(6280), 1450–1454.

[14] Jolma, A., Yan, J., Whitington, T., Toivonen, J., Nitta, K. R., Rastas, P., Morgunova, E., Enge, M., Taipale, M., Wei, G., Palin, K., Vaquerizas, J. M., Vincentelli, R., Luscombe, N. M., Hughes, T. R., Lemaire, P., Ukkonen, E., Kivioja, T., and Taipale, J. (jan, 2013) DNA-binding specificities of human transcription factors. *Cell,* **152**(1-2), 327–339.

[15] Nitta, K. R., Jolma, A., Yin, Y., Morgunova, E., Kivioja, T., Akhtar, J., Hens, K., Toivonen, J., Deplancke, B., Furlong, E. E., and Taipale, J. (2015) Conservation of transcription factor binding specificities across 600 million years of bilateria evolution. *eLife,* **2015**(4), doi: 10.7554/eLife.04837.

[16] Yin, Y., Morgunova, E., Jolma, A., Kaasinen, E., Sahu, B., Khund-Sayeed, S., Das, P. K., Kivioja, T., Dave, K., Zhong, F., Nitta, K. R., Taipale, M., Popov, A., Ginno, P. A., Domcke, S., Yan, J., Schübeler, D., Vinson, C., and Taipale, J. (2017) Impact of cytosine methylation on DNA binding specificities of human transcription factors. *Science,* **356**(6337).

[17] Zhu, L. J., Christensen, R. G., Kazemian, M., Hull, C. J., Enuameh, M. S., Basciotta, M. D., Brasefield, J. A., Zhu, C., Asriyan, Y., Lapointe, D. S., Sinha, S., Wolfe, S. A., and Brodsky, M. H. (2011) FlyFactorSurvey: A database of Drosophila transcription factor binding specificities determined using the bacterial one-hybrid system. *Nucleic Acids Research,* **39**(SUPPL. 1), D111–D117.

[18] Busser, B. W., Shokri, L., Jaeger, S. A., Gisselbrecht, S. S., Singhania, A., Berger, M. F., Zhou, B., Bulyk, M. L., and Michelson, A. M. (2012) Molecular mechanism underlying the regulatory specificity of a Drosophila homeodomain protein that specifies myoblast identity. *Development,* **139**(6), 1164–1174.

[19] Noyes, M. B., Christensen, R. G., Wakabayashi, A., Stormo, G. D., Brodsky, M. H., and Wolfe, S. A. (jun, 2008) Analysis of Homeodomain Specificities Allows the Family-wide Prediction of Preferred Recognition Sites. *Cell,* **133**(7), 1277–1289.

[20] Bateman, A. (2019) UniProt: A worldwide hub of protein knowledge. *Nucleic Acids Research,* **47**(D1), D506–D515.

[21] Sonnhammer, E. L. L., Eddy, S. R., and Durbin, R. (1997) Pfam: A comprehensive database of protein domain families based on seed alignments. *Proteins: Structure, Function and Genetics,* **28**(3), 405–420.

[22] Wolfe, S. A., Nekludova, L., and Pabo, C. O. (2000) DNA Recognition by Cys2His2 Zinc Finger Proteins. *Annual Review of Biophysics and Biomolecular Structure,* **29**(1), 183–212.

[23] Chu, S. W., Noyes, M. B., Christensen, R. G., Pierce, B. G., Zhu, L. J., Weng, Z., Stormo, G. D., and Wolfe, S. A. (2012) Exploring the DNA-recognition potential of homeodomains. *Genome Research,* **22**(10), 1889–1898.

3 Supplemental Figures (next page)

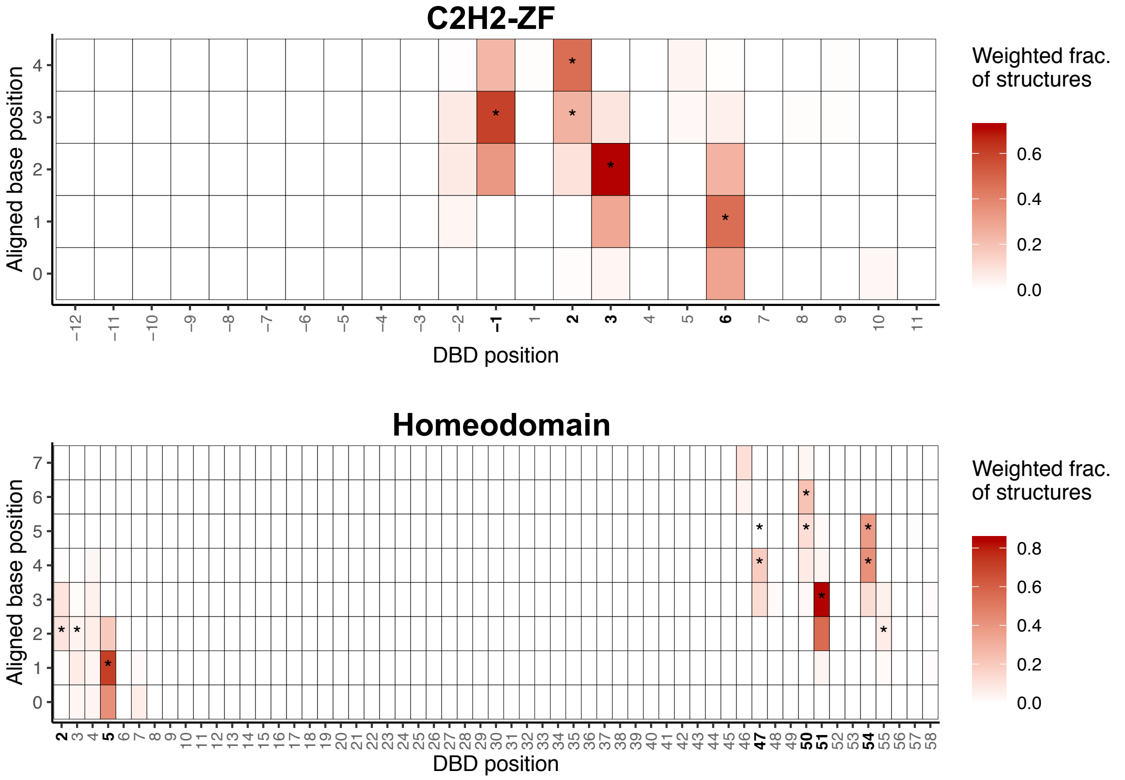

Figure S1: **Contact frequency matrices inferred from co-complex structural data.** For each of two DBD families, C2H2-ZFs (top) and Homeodomains (bottom), we display a heat map representation of the contact frequency matrix inferred by applying our algorithm across the set of protein-DNA co-complex structures found in BioLiP [1] for that DBD family (Supplemental Methods 1.1 and 1.2). Each grid's horizontal axis corresponds to a DBD position, known based on PFAM HMM match states (labeled according to the "canonical" numbering scheme for the DBD family), while its vertical axis corresponds to a base position numbered relative to the start of the "core" binding site (position 1), with one additional position shown flanking either side of the core binding site. The color in each cell represents the uniqueness-weighted fraction of DBD-DNA interfaces in which a DNA base in a given (aligned) base position contacts (defined as at most 3.6 Å distance between heavy atoms) an amino acid side chain in a given DBD position. Bolded DBD positions are considered as "base-contacting" for the purpose of our analyses, in that they contact a particular base position within the core binding site in at least 10% of uniqueness-weighted DBD-DNA interfaces. DBD-base position pairs marked with asterisks correspond to contacts proposed in previous literature [22, 19, 23] to be important specificity determinants, via either structural analyses or correlation of residue and base identities in combination.

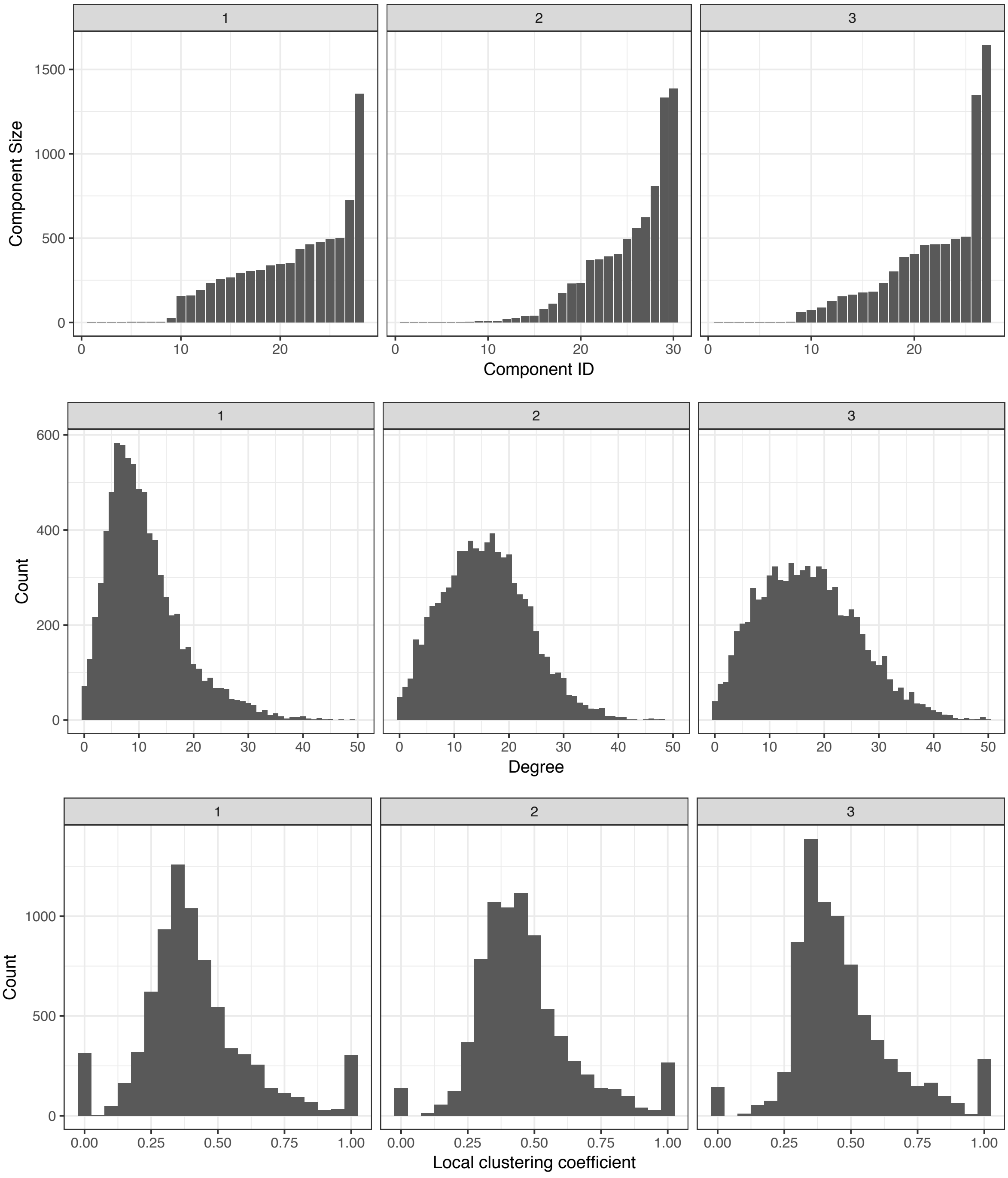

Figure S2: **Properties of similarity graphs from `PW-2015` dataset.** For similarity graphs (see Methods) corresponding to base positions 1, 2, and 3 (left, middle, and right, respectively) for the `PW-2015` dataset, we display as histograms the distributions of connected component sizes (top), degree (middle), and local clustering coefficients (bottom).

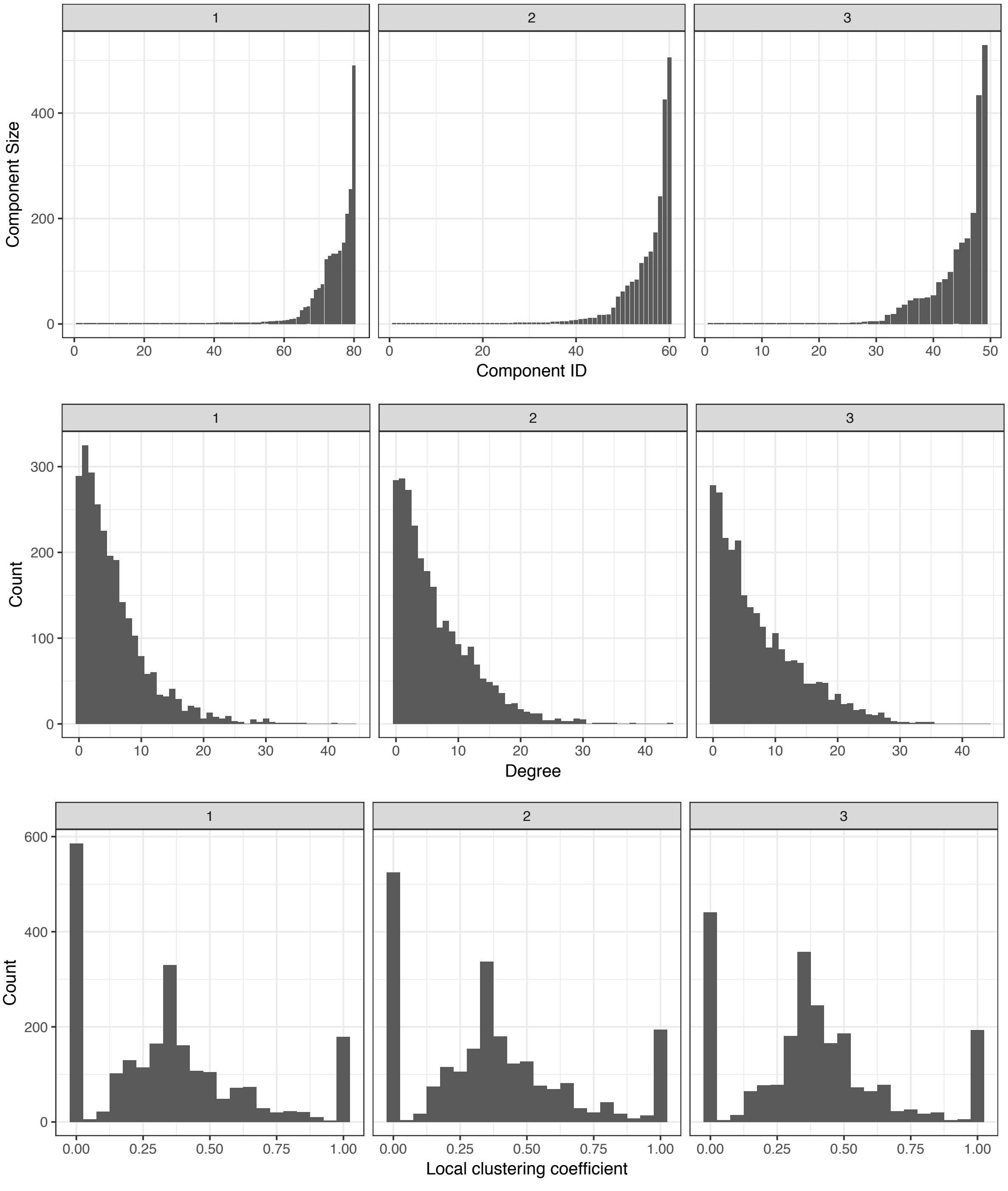

Figure S3: **Properties of similarity graphs from `NM-2015` dataset.** For similarity graphs (see Methods) corresponding to base positions 1, 2, and 3 (left, middle, and right, respectively) for the `NM-2015` dataset, we display as histograms the distributions of connected component sizes (top), degree (middle), and local clustering coefficients (bottom).

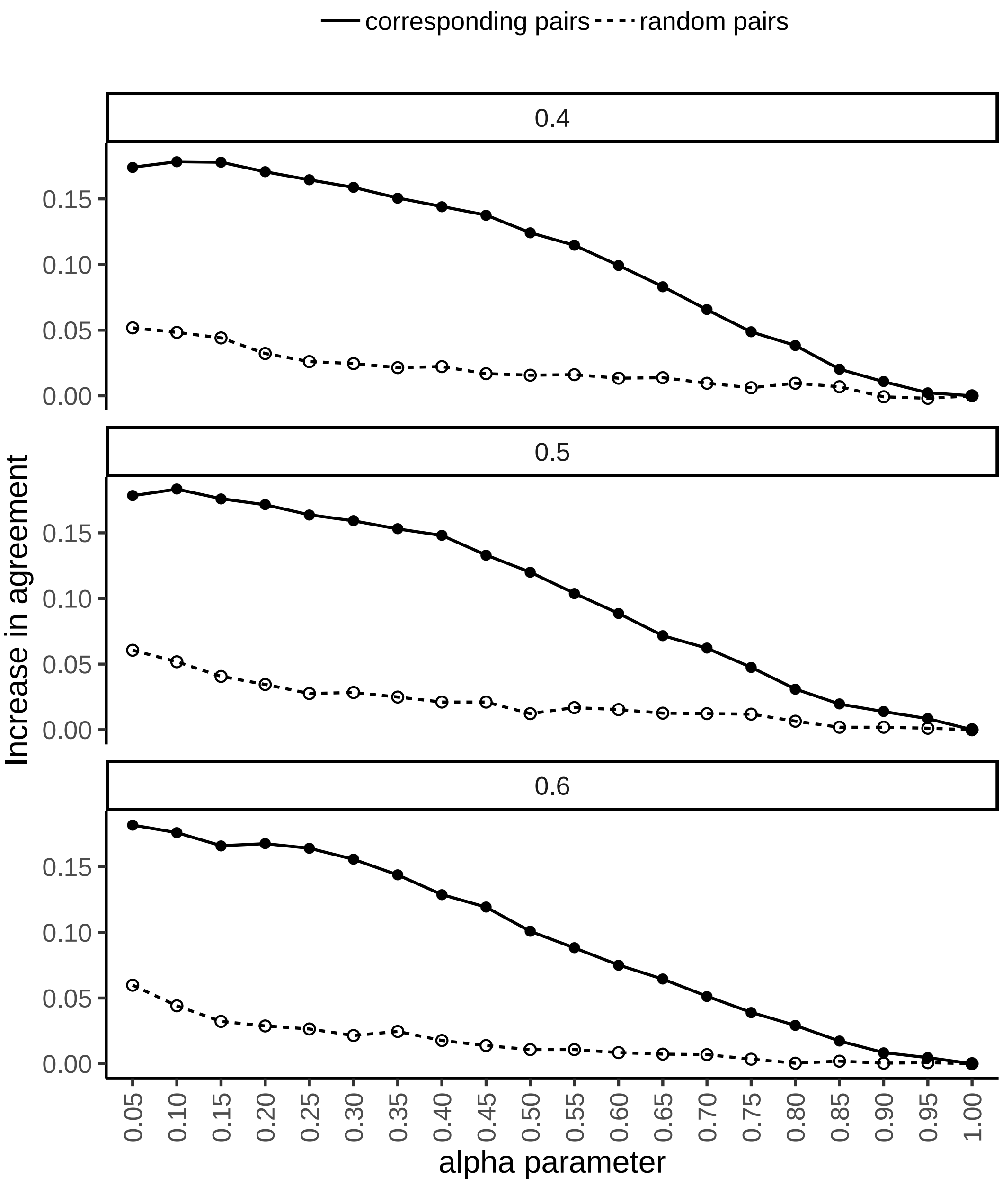

Figure S4: **Increases in across-dataset agreement are robust to changes in the PCC agreement threshold.** We apply the QP formulation to each dataset separately for different values of α. As described in Figure 1 top of the main manuscript, across a range of α settings (x-axis), we compare across-dataset agreement increase (y-axis) across all PWM columns for corresponding core sequence pairs between the `NM-2015` and `PW-2015` datasets (solid lines) and for random across-dataset core sequence pairs (dashed lines). Overall, our results are similar when considering PCC agreement thresholds of 0.4 (top), 0.5 (middle; shown also in Figure 1 top), or 0.6 (bottom).

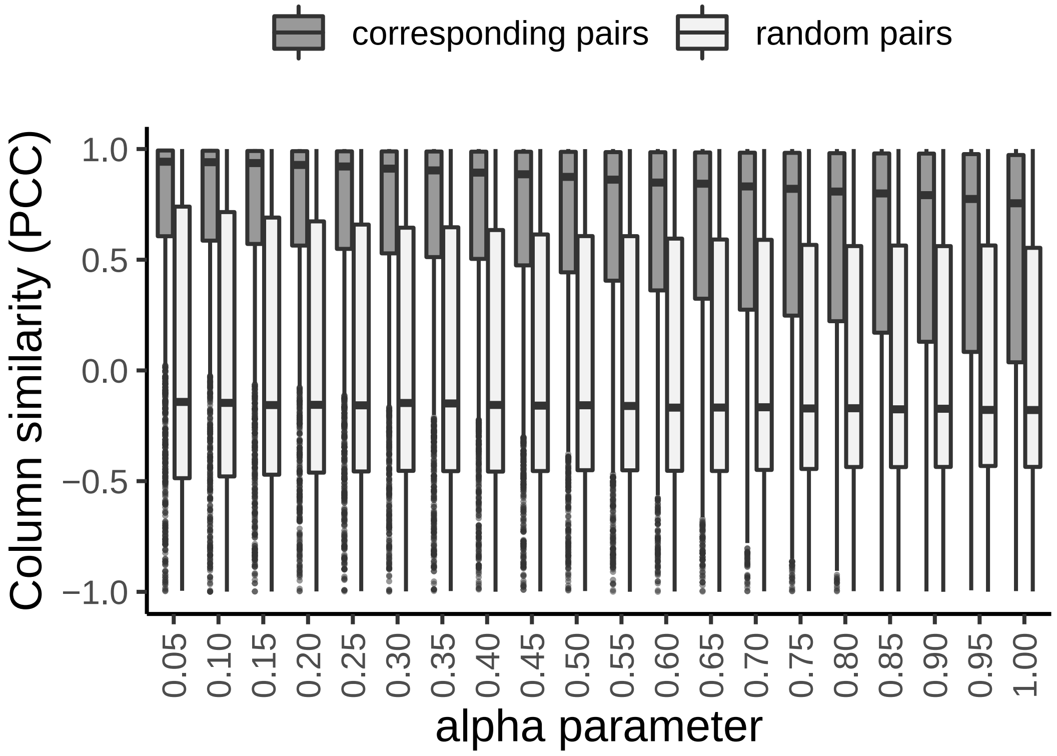

Figure S5: **Distributions of across-dataset agreement scores.** We apply the QP formulation to each dataset separately for different values of α. For each α setting (x-axis), we consider agreement score distributions (PCC, y-axis) for corresponding column pairs across the `NM-2015` and `PW-2015` datasets (dark gray), or for random across-dataset column pairs (light gray). We depict each distribution as a boxplot where whiskers extend to 1.5x the interquartile range. Overall, we observe an increase in median agreement and a decrease in variance as α moves from 1 to 0.05 for the corresponding column pairs. For random pairs, the medians remain similar across the α range, with increases in variance as α becomes small. Notably, at low α, for random pairs, there is a marked increase in the third quartile, corresponding to an increase in the number of random pairs with high PCC.

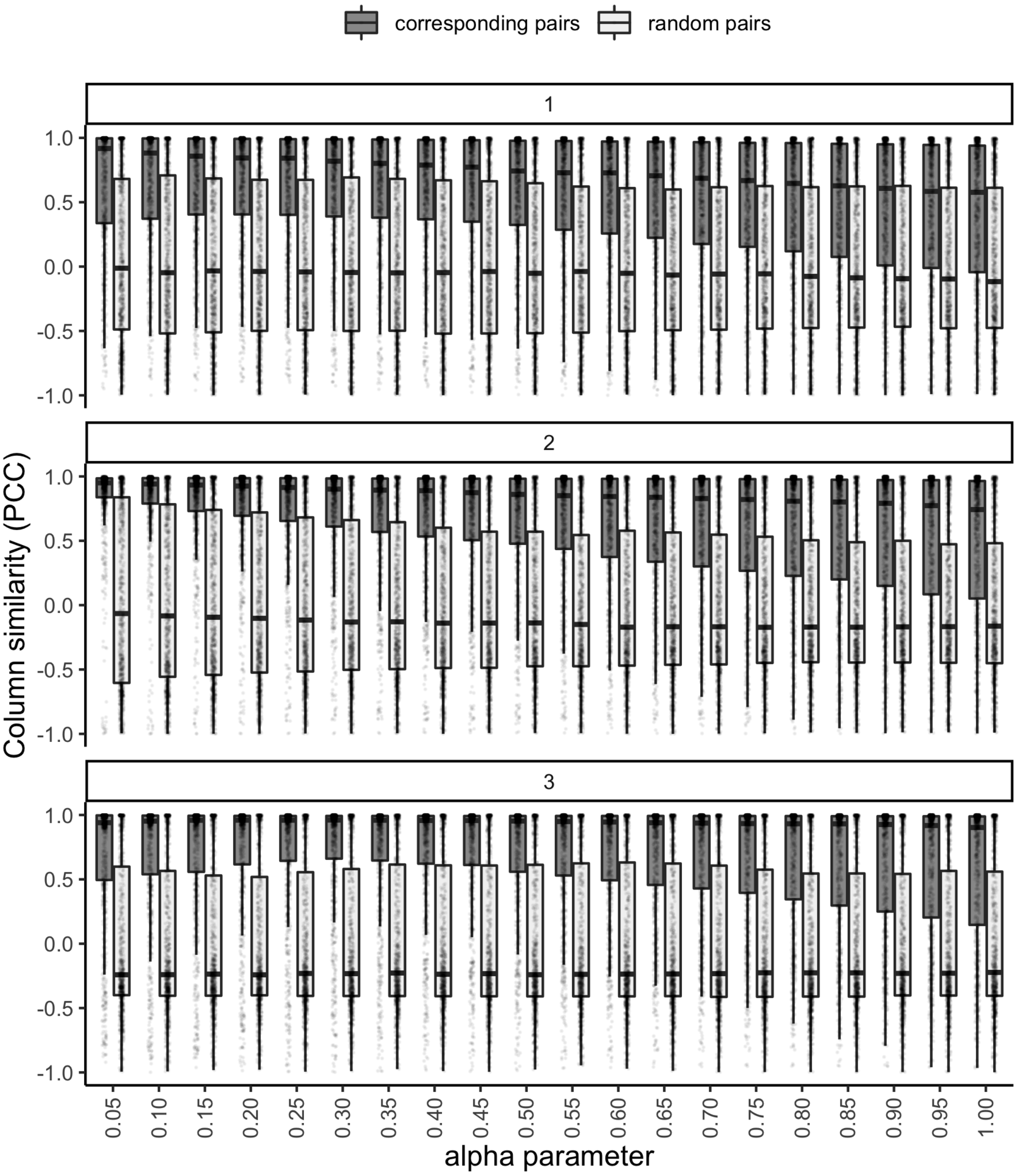

Figure S6: **Distributions of across-dataset agreement scores, per base positon.** We apply the QP formulation to each dataset separately for different values of α. As in Supplemental Figure S5, we consider agreement score distributions (PCC, y-axis) for corresponding column pairs across the `NM-2015` and `PW-2015` datasets (dark gray), or for random across-dataset column pairs (light gray) at each α setting (x-axis), partitioning scores for column pairs of bases in positions 1 (top), 2 (middle), or 3 (bottom) of the PWMs. We depict each distribution as a boxplot where whiskers extend to 1.5x the interquartile range, with individual data points overlaid. Trends are overall similar to when considering scores form all base positions in aggregate (see Supplemental Figure S5).

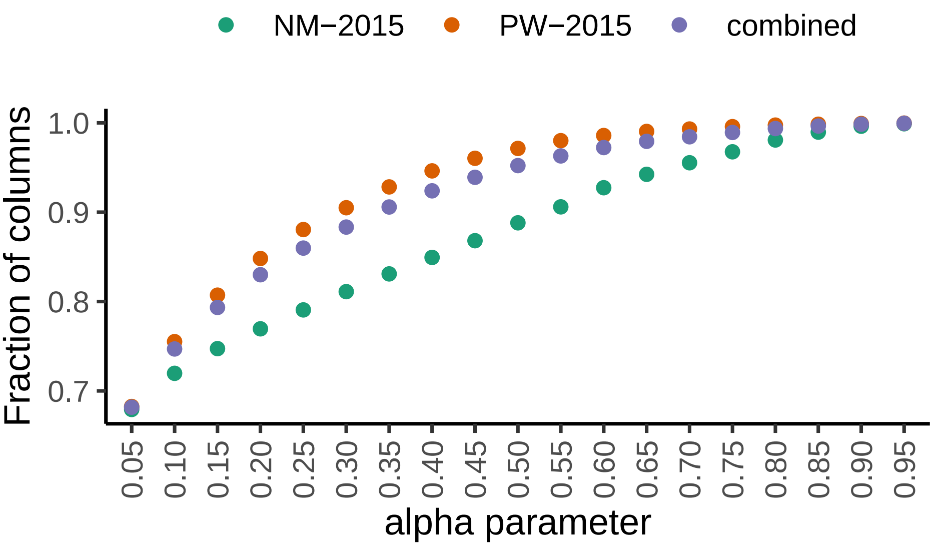

Figure S7: **Jointly inferred specificities tend to agree with initial specificities.** Considering specificities for the `NM-2015` and `PW-2015` datasets separately (green and red, respectively) or in aggregate (purple), we plot the fraction of columns that remain in agreement with their initial counterparts (y-axis) after applying the QP procedure, as a function of the α parameter (x-axis). At reasonable α settings, the inferred and initial specificities are usually in agreement; e.g., across all combined columns at $\alpha = 0.4$, 92% remain in agreement with their initial counterparts.

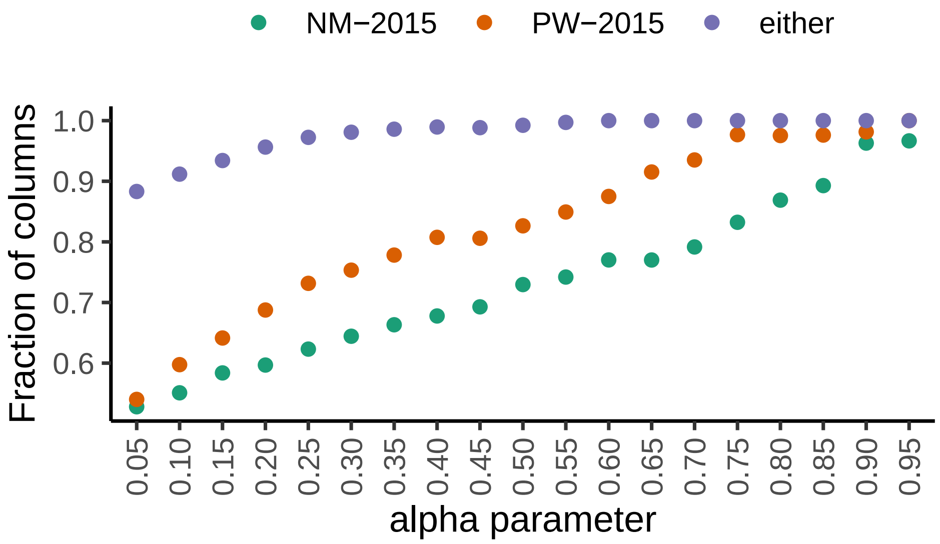

Figure S8: **When corresponding PWM column pairs gain agreement across datasets, at least one column in the pair tends to remain in agreement with its initial counterpart.** Considering the subset of PWM columns that gain agreement after application of the QP procedure (i.e., the corresponding jointly inferred column pairs agree, but their initial counterparts do not; as described in Figure 2 of the main manuscript), we plot, for the `NM-2015` and `PW-2015` datasets separately (green and red, respectively) the fraction of inferred columns that remain in agreement with their initial counterparts (y-axis), as a function of the α parameter (x-axis). At each α, considering each corresponding column pair across the two datasets, we also plot the fraction of the time that at least one column from the pair remains in agreement with its initial counterpart ('either'; purple). For example, when considering all corresponding column pairs at $\alpha = 0.4$, at least one column from the pair remains in agreement with its initial counterpart 99% of the time.

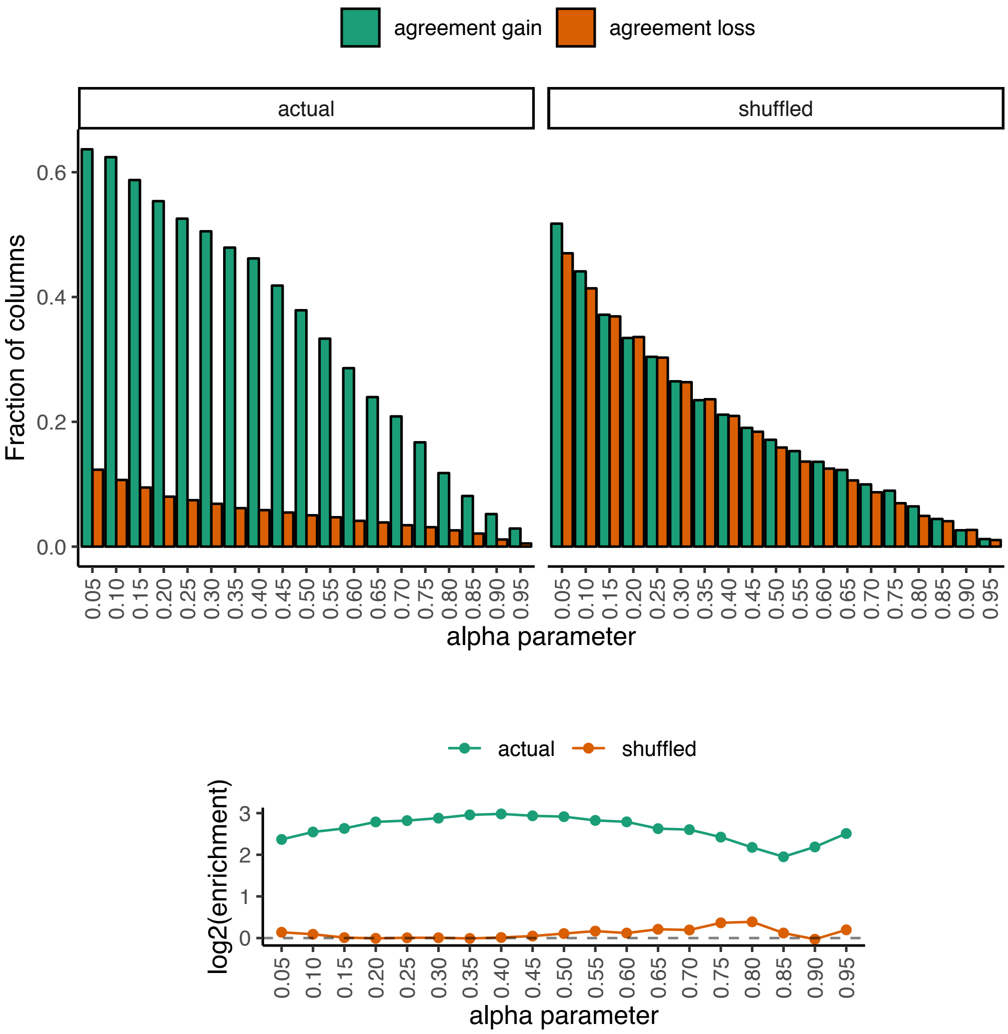

Figure S9: **Ratio of across-dataset agreement gain to agreement loss under actual versus shuffled within-dataset core sequence associations.** For different values of α, we apply the QP formulation to each dataset separately (top left, 'actual') and on randomized data obtained by shuffling PWM columns within each similarity graph to randomize core sequence pair relationships within each dataset (top right, 'shuffled'). As in Figure 2 of the main manuscript, we consider the trade-off between agreement gain and agreement loss for corresponding column pairs across the `NM-2015` and `PW-2015` datasets, when varying the regularization parameter α (x-axis). (top) For each α, we plot the fraction of initially disagreeing columns that agree (green; y-axis) and the fraction of initially agreeing columns that disagree (red; y-axis). (bottom) We plot the logarithm of the ratios of the two values (agreement gain over agreement loss; y-axis) at each α setting under the actual (green line) or the shuffled (red line) core sequence associations. While there is clear enrichment for agreement gain under the actual core sequence associations, the ratio remains close to 1 under the shuffled core sequence associations across all α settings.

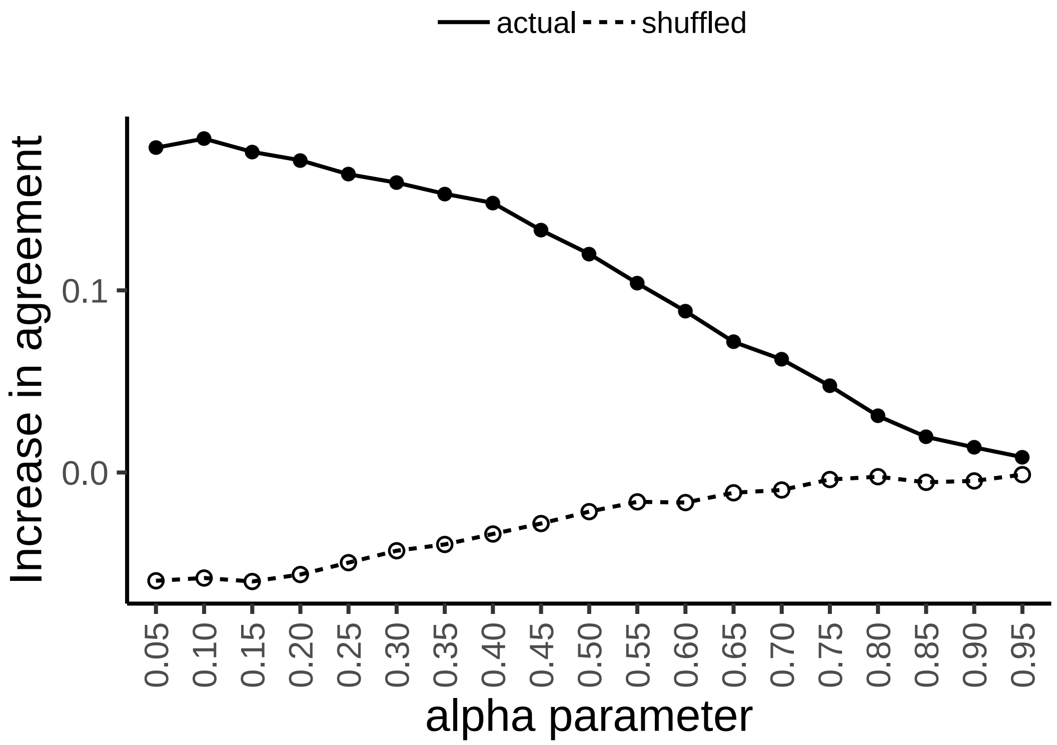

Figure S10: **Across-dataset agreement decreases when within-dataset core sequence associations are shuffled.** Similarly to Figure 1 top, we plot the increase in fraction of corresponding columns in across-dataset agreement for `NM-2015` and `PW-2015` (i.e., fraction of columns in agreement at α minus fraction of columns initially agreeing) when using QP with actual (solid line) versus shuffled (described in Supplemental Figure S9; dashed line) core sequence relationships within each dataset. While there is clear gain of across-dataset agreement under the actual core sequence associations, across-dataset agreement decreases under the shuffled core sequence associations.

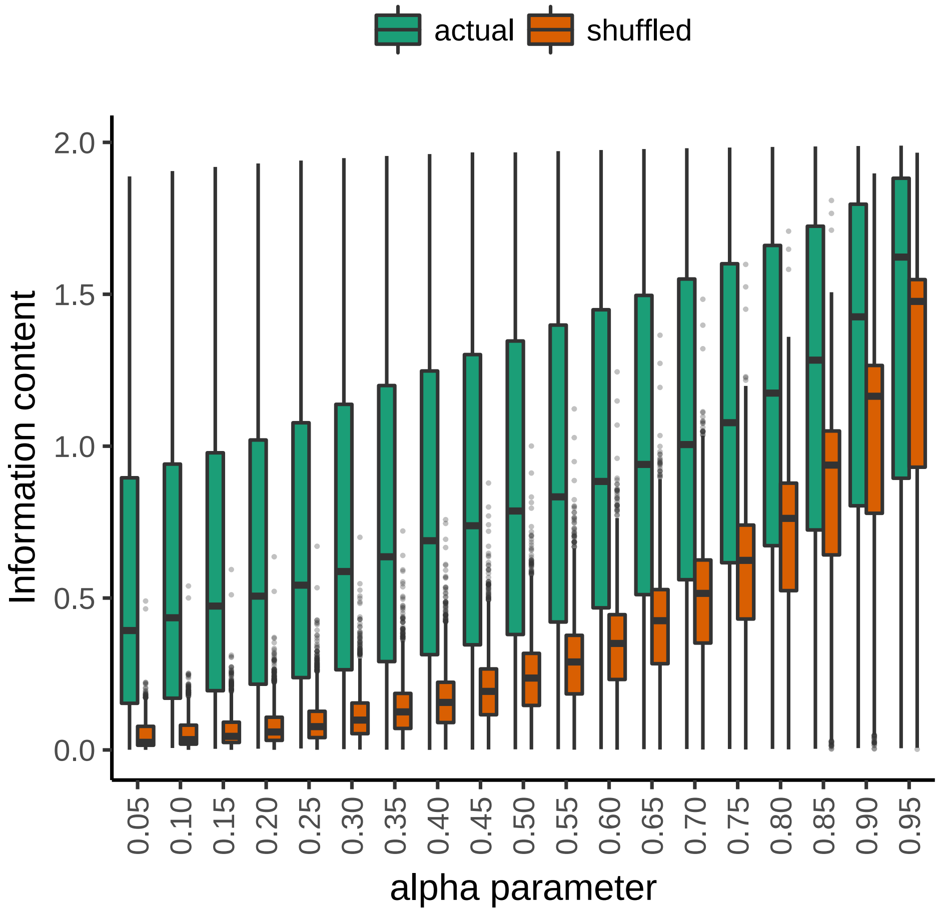

Figure S11: **PWM columns determined using shuffled within-dataset core sequence associations lack information content.** Considering all jointly inferred PWM columns of core sequences present in both `PW-2015` and `NM-2015`, we visualize as boxplots the distributions of information content (IC) of columns (using the maximum IC across each corresponding column pair across datasets) determined using QP under either actual (green) or shuffled (described in Supplemental Figure S9; red) within-dataset core sequence relationships, at each α setting (x-axis). Under the shuffled relationships there is pronounced loss of IC, especially at small α, where PWM columns tend to contain almost no information; this is substantially lower than IC for columns determined using actual within-dataset core sequence relationships. For each boxplot, whiskers extend to 1.5x the interquartile range.

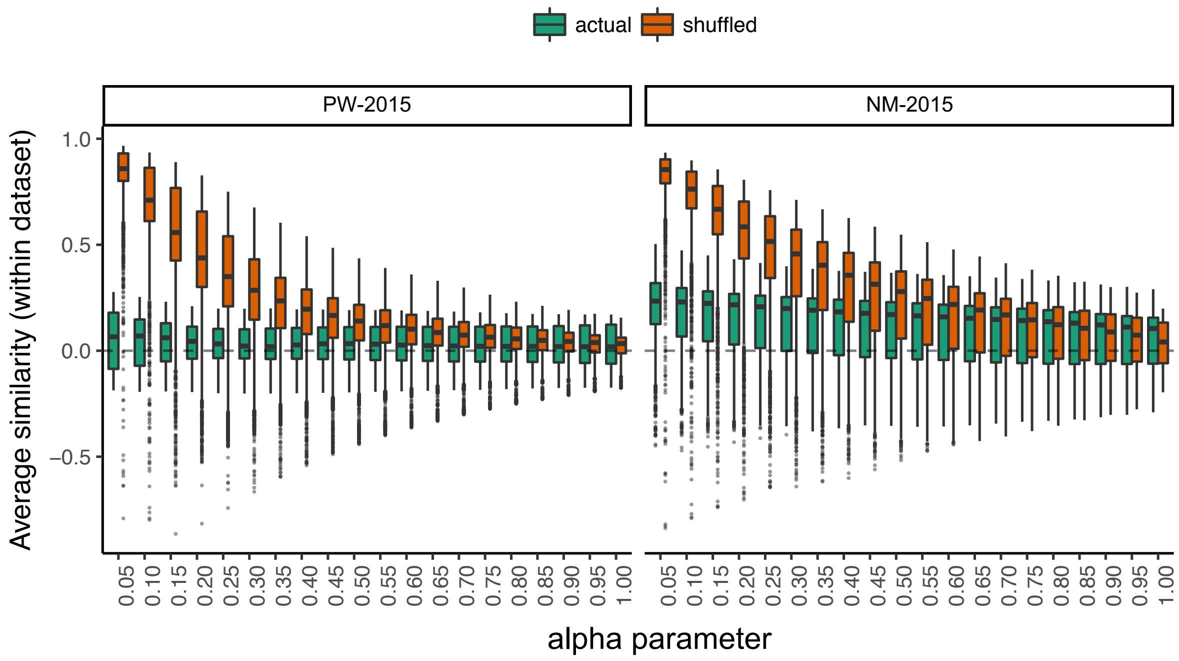

Figure S12: **PWM columns determined using shuffled within-dataset core sequence associations lack diversity.** We visualize within-dataset PWM column diversity considering all QP jointly inferred PWM columns of core sequences present in both `PW-2015` (left) and `NM-2015` (right) under actual (green) versus shuffled (described in Supplemental Figure S9; red) within-dataset core sequence associations. At each α level (x-axis), we compute the average similarity (PCC; y-axis) of each jointly inferred column to every other jointly inferred column within the same dataset and display the distributions of these average similarity scores as boxplots. For both `PW-2015` and `NM-2015`, jointly inferred specificities under the shuffled associations tend to become highly similar to one another as α decreases, while jointly inferred specificities under the actual associations tend to maintain their diversity. For each boxplot, whiskers extend to 1.5x the interquartile range.

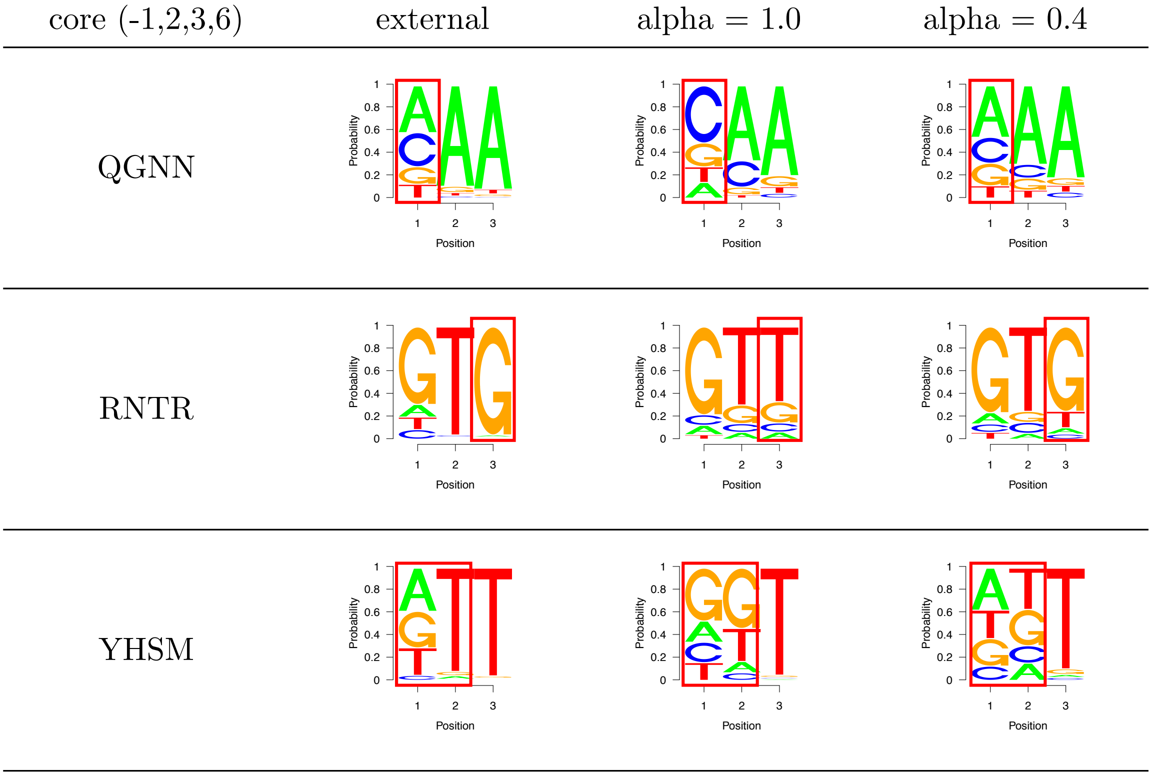

Figure S13: **Examples of improved agreement for jointly inferred C2H2-ZF PWMs.** For three sequence-diverse C2H2-ZF core sequences present in both the `NM-2015` dataset and the external dataset [9], we show frequencies before and after applying joint PWM inference (QP). For each core sequence, we show the external PWM from Enuameh et al. [9] (left; "external"), the initial PWM from `NM-2015` ($\alpha = 1$; middle), and the PWM jointly inferred using `NM-2015` with $\alpha = 0.4$ (right). Red boxes highlight base positions for which the initial PWM was in disagreement with the external standard, but is in agreement with it at $\alpha = 0.4$.

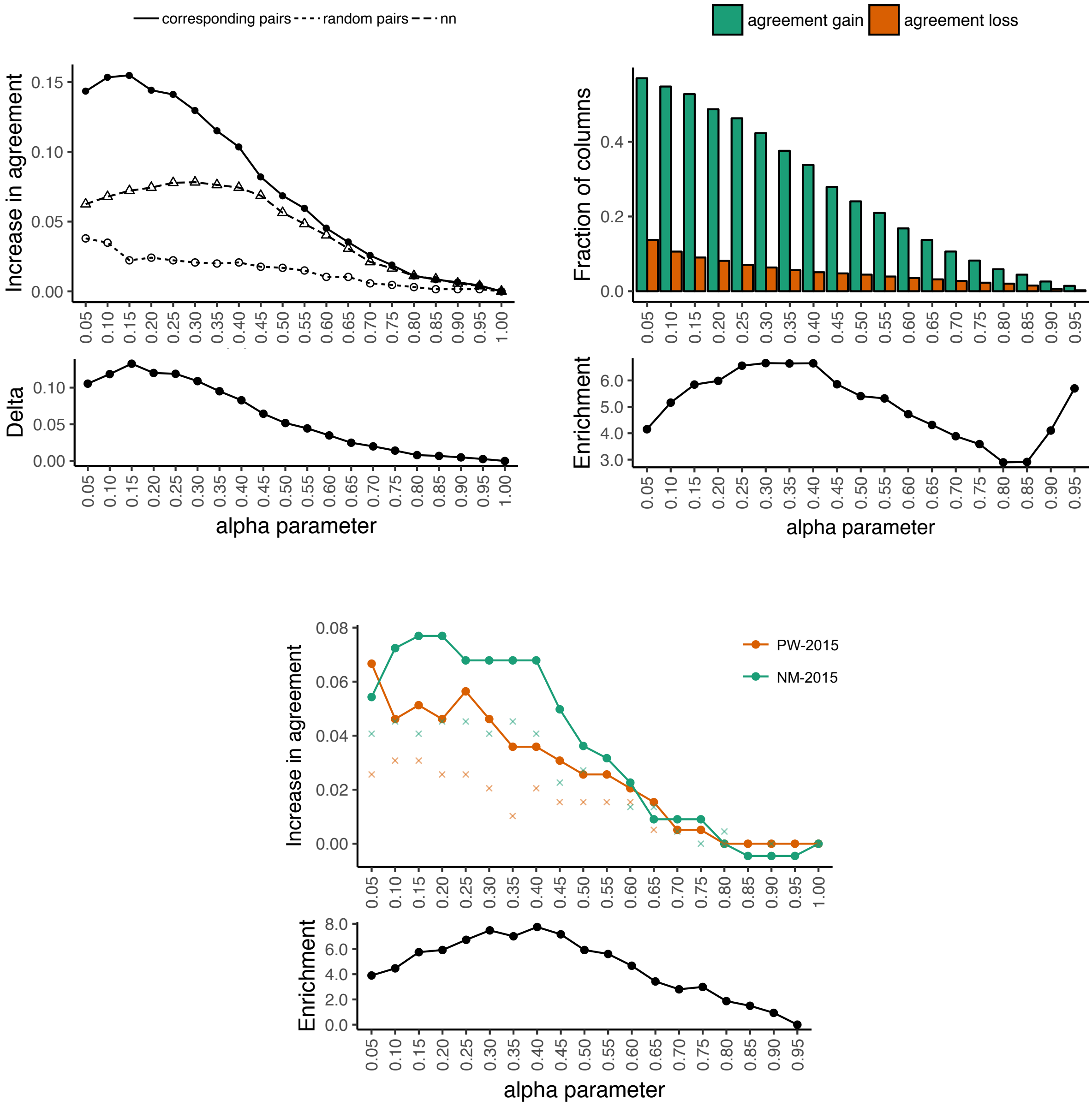

Figure S14: **Similar trends are observed when using label propagation adsorption (LPA) as when using quadratic programming (QP).** We repeated all of the analyses described in the main manuscript for the QP formulation, replacing it with LPA, observing overall similar trends. Here we show figures analogous to main manuscript Figures 1 (top left), 2 (top right), and 3 (bottom), but using LPA. For the plots analogous to Figures 1 and 3, we have additionally included points to display the increase in across-dataset agreement when using a nearest neighbors approach ('nn'; triangles and crosses for Figures 1 and 3, respectively) based on a single iteration of the LPA algorithm (as described in Results). For nearly all α, LPA to convergence outperforms the nearest neighbors approach, demonstrating the benefits of rewarding global, rather than simply local, pairwise consistency of specificities under consideration of the same structurally derived protein similarity measure.

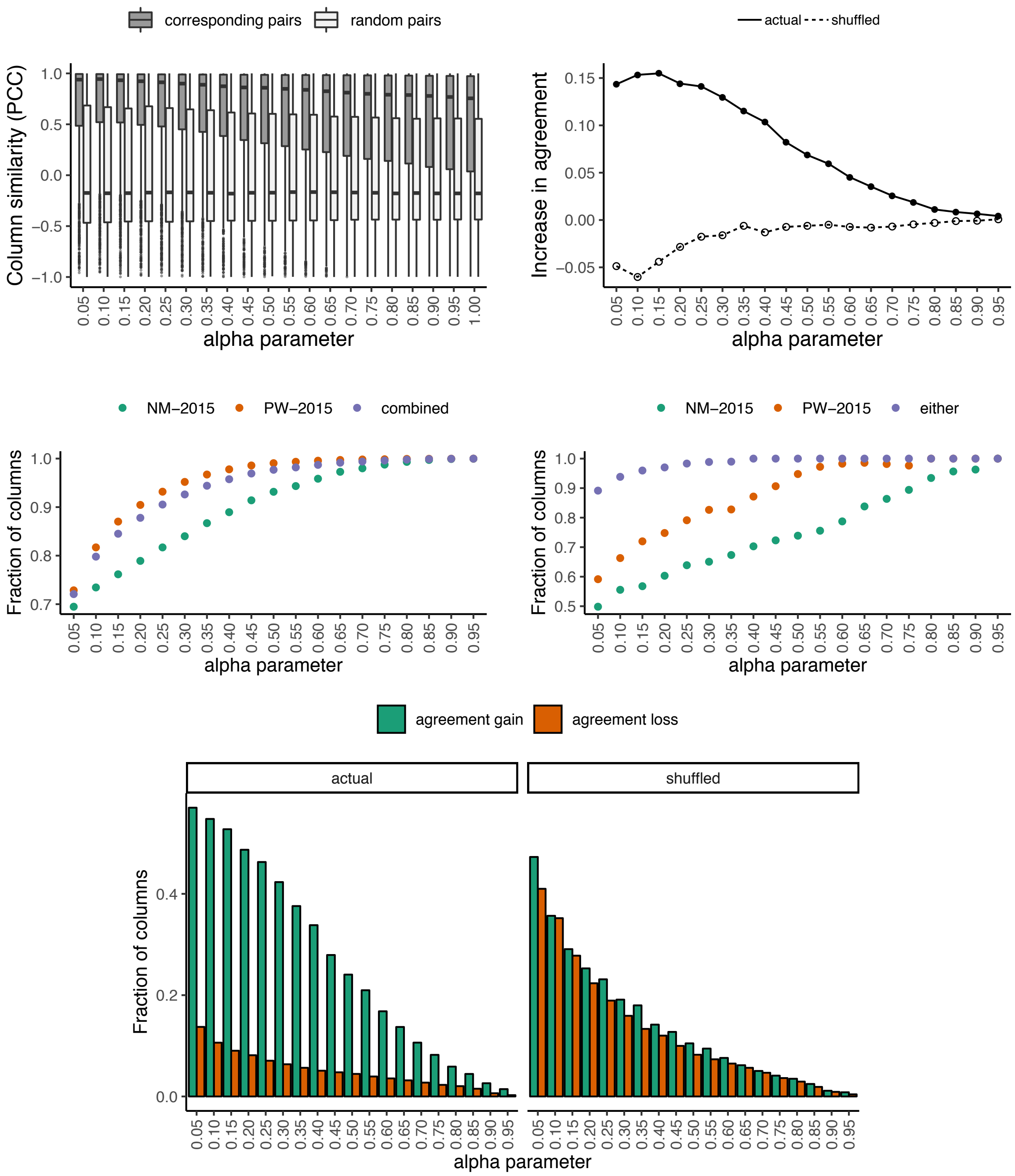

Figure S15: **Similar trends are observed when using label propagation adsorption (LPA) as when using quadratic programming (QP).** We repeated all of the analyses described in the main manuscript for the QP formulation, replacing it with the LPA, observing overall similar trends. Here we show figures analogous to Supplemental Figures S5 (top left), S10 (top right), S7 (middle left), S8 (middle right) and S9 top (bottom).

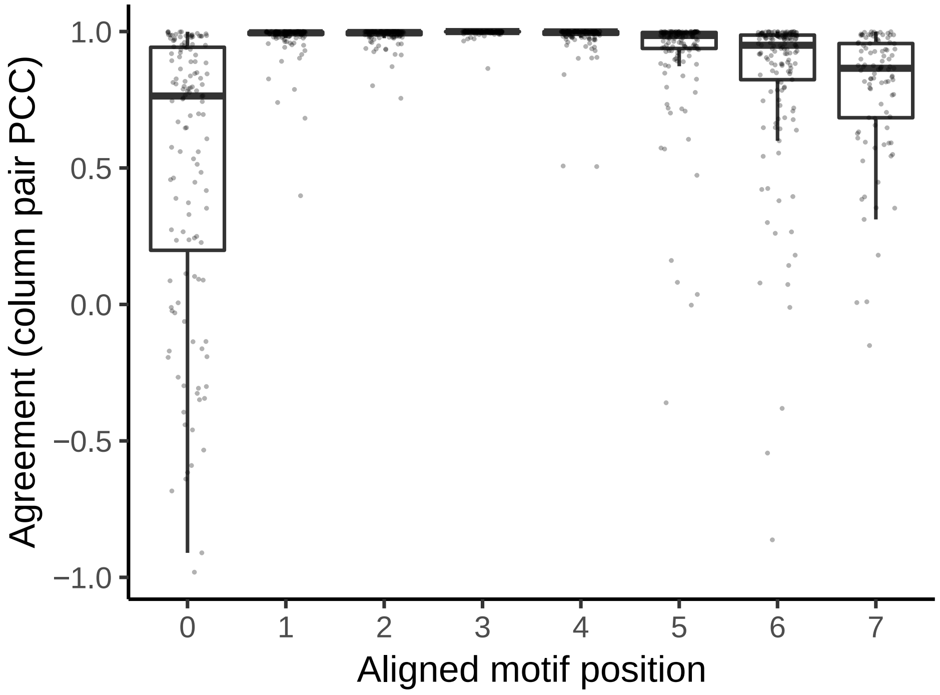

Figure S16: **Pairwise agreement for aligned Homeodomain PWMs from independent publications.** For 83 proteins where two or more PWMs from independent publications existed, we plot the distributions of agreement scores (PCC; y-axis) for all PWM column pairs corresponding to the same binding site position and the same protein. We depict each distribution of agreement scores as a boxplot where whiskers extend to 1.5x the interquartile range, with the individual data points overlaid. The base positions are numbered according a canonical numbering scheme where positions 1 through 6 correspond to the six positions of the "core" HD binding site (i.e., positions 1 through 4 correspond to the `TAAT` motif shared across proteins of the Antp and En HD subfamilies). For these four positions, we observe excellent agreement across nearly all of the corresponding column pairs, while for positions 5 and 6 we observed several disagreeing pairs (i.e, PCC < 0.5). As expected, flanking positions 0 and 7 show the lowest level of agreement, as they contribute only weakly to specificity and the mechanism of their recognition by the HD is poorly understood.

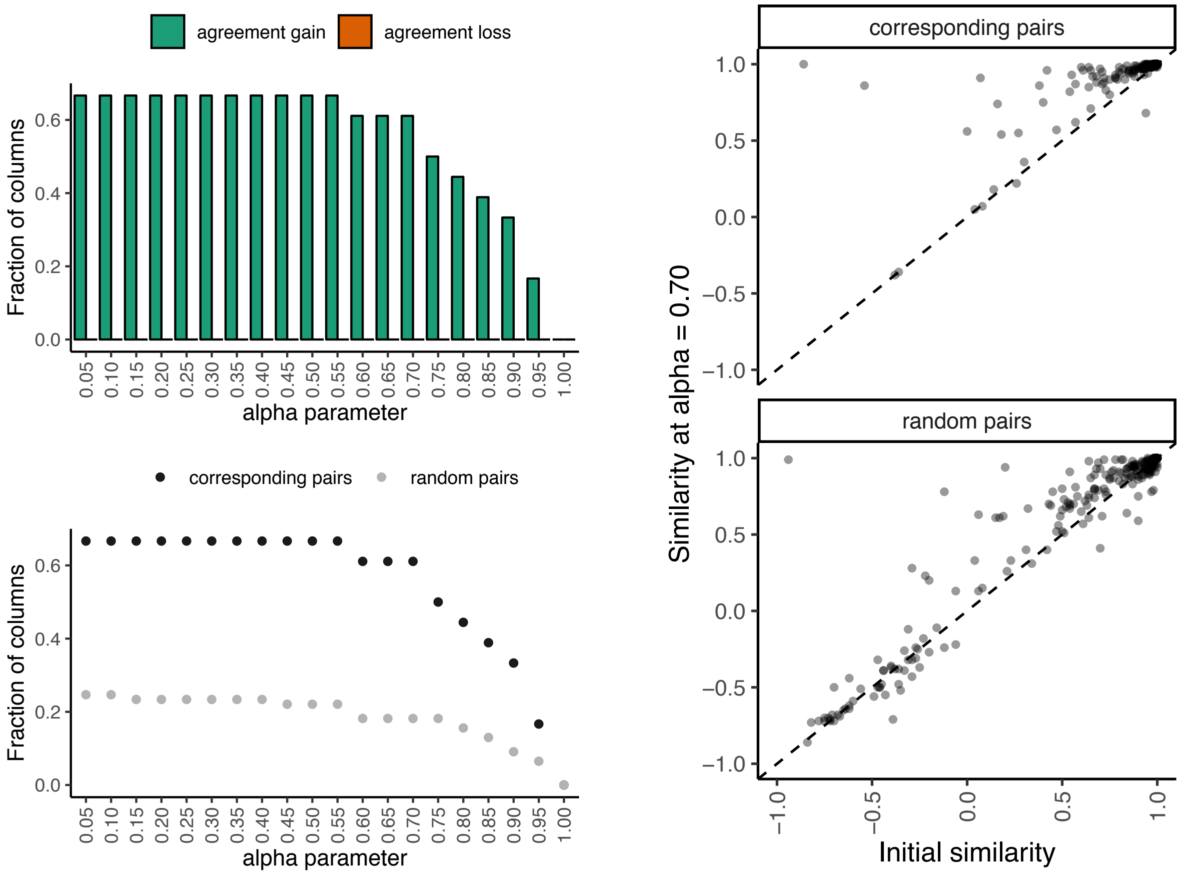

Figure S17: **Joinly inferring specificities for Homeodomain proteins via the QP approach improves agreement for positions 5 and 6 across PWMs from independent publications.** 429 oriented and aligned HD PWMs (Supplemental Methods 1.4 and 1.5) were partitioned into two sets of roughly equal size, and our QP approach was applied to base positions 5 and 6 for each set independently using a pairwise protein similarity measure based on structural considerations, and tested at various α settings (Supplemental Methods 1.6 and Supplemental Results 2.1). (top, left) We consider agreement gain and agreement loss for column pairs corresponding to the same position and same protein, but in opposite sets. Specifically, we compare the jointly inferred specificities in each of the two sets and compute the fraction (y-axis) of initially disagreeing columns that now agree (green) and the fraction of initially agreeing columns that now disagree (red). Of the column pairs that initially disagreed, $\geq$61% of them are in agreement for all $\alpha \leq 0.7$, while none of the columns that initially agreed go into disagreement by sharing information. (bottom, left) As a control, we consider agreement gain at each α setting for columns randomly paired (within each base positions) across the two sets. For all $\alpha \leq 0.95$ tested, agreement gain (y-axis) is higher for corresponding pairs than for random pairs (black and gray dots, respectively), indicating that the level of agreement gain observed for corresponding pairs cannot be explained by protein-independent similarity of background per-base-position nucleotide compositions across the two sets. For example, at $\alpha = 0.7$, 61% of initially disagreeing columns pairs have moved into agreement, while the same statistic for random pairs is only 18%. (right) For each corresponding and each random column pair (top and bottom, respectively), we compare the initial column similarity (PCC; x-axis) to the column similarity when using QP with $\alpha = 0.7$ (PCC; y-axis). We observe that the QP procedure rarely decreases correlation for corresponding pairs (only 3% to right of diagonal), while 28% of the random pairs decreased in correlation.

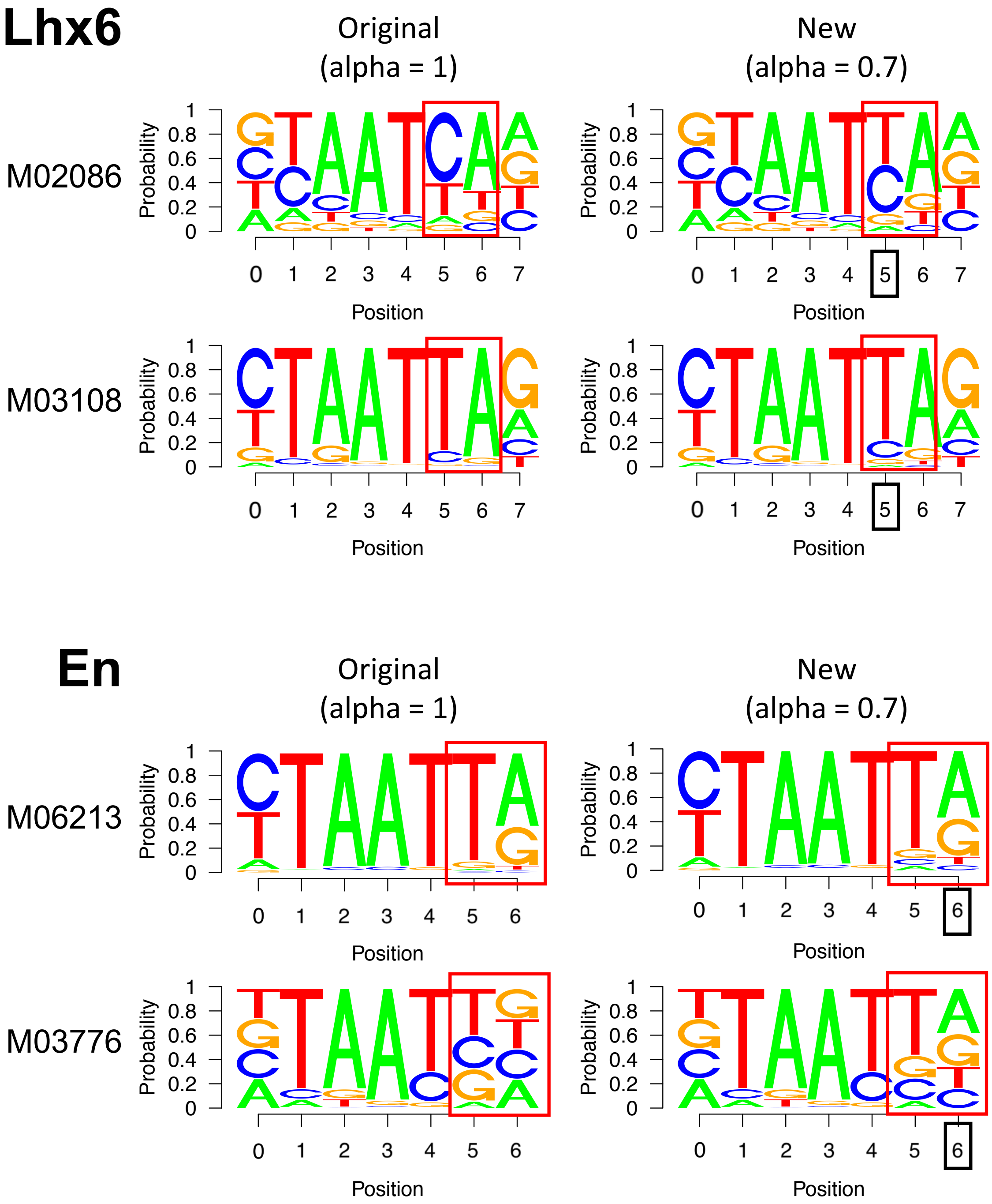

Figure S18: **Examples of improved agreement for Homeodomain PWMs from independent publications.** Frequency logos for PWMs of two transcription factors, Lhx6 (top, Cis-BP ID T209796) and En (bottom, Cis BP ID T217414), are given before (left; 'original'; $\alpha = 1$) and after (right; 'new'; $\alpha = 0.7$) applying our QP procedure for base positions 5 and 6 (red boxed positions) independently to two sets of HD PWMs (as described in Supplemental Results 2.1). (top) For Lhx6, we show two PWMs (`M02086` and `M03108`) from opposite sets that are originally in poor agreement in base position 5, but are in good agreement after using the QP procedure with $\alpha = 0.7$, both displaying a preference for thymine over cytosine in this position. (bottom) For En, we show two PWMs (`M06213` and `M03776`) from opposite sets that originally disagreed at position 6, but agree after running the QP procedure using $\alpha = 0.7$, each displaying a preference for adenine or guanine.
